# Genome‐wide patterns of homoeologous gene flow in allotetraploid coffee

**DOI:** 10.1002/aps3.11584

**Published:** 2024-06-14

**Authors:** Andre J. Ortiz, Joel Sharbrough

**Affiliations:** ^1^ Department of Biology New Mexico Institute of Mining and Technology Socorro New Mexico USA

**Keywords:** ABBA‐BABA test, allopolyploidy, chloroplast, *Coffea arabica*, homoeologous gene flow, mitochondria, whole‐genome duplication

## Abstract

**Premise:**

Allopolyploidy—a hybridization‐induced whole‐genome duplication event—has been a major driver of plant diversification. The extent to which chromosomes pair with their proper homolog vs. with their homoeolog in allopolyploids varies across taxa, and methods to detect homoeologous gene flow (HGF) are needed to understand how HGF has shaped polyploid lineages.

**Methods:**

The ABBA‐BABA test represents a classic method for detecting introgression between closely related species, but here we developed a modified use of the ABBA‐BABA test to characterize the extent and direction of HGF in allotetraploid *Coffea arabica*.

**Results:**

We found that HGF is abundant in the *C. arabica* genome, with both subgenomes serving as donors and recipients of variation. We also found that HGF is highly maternally biased in plastid‐targeted—but not mitochondrial‐targeted—genes, as would be expected if plastid–nuclear incompatibilities exist between the two parent species.

**Discussion:**

Together, our analyses provide a simple framework for detecting HGF and new evidence consistent with selection favoring overwriting of paternally derived alleles by maternally derived alleles to ameliorate plastid–nuclear incompatibilities. Natural selection therefore appears to shape the direction and intensity of HGF in allopolyploid coffee, indicating that cytoplasmic inheritance has long‐term consequences for polyploid lineages.

Whole‐genome duplication events (WGDs), in which the entire nuclear genome is doubled resulting in polyploid lineages, are widespread among plants, especially angiosperms (Jiao et al., 2011; Wendel, 2015; Ruprecht et al., 2017; One Thousand Plant Transcriptomes Initiative, 2019; Román‐Palacios et al., 2021; Heslop‐Harrison et al., 2023). WGDs are among the most profound mutational changes observed in nature because they result in global genomic redundancy, which has consequences that range from the gene to the population level (Fox et al., 2020). At the gene level, genomic redundancy contributes to relaxation of selective constraints (Otto and Whitton, 2000; Douglas et al., 2015; Zhang et al., 2020; Conover and Wendel, 2022), transcriptional reprogramming (Schnable et al., 2011; Combes et al., 2013; Yoo et al., 2013; Akama et al., 2014; Hu et al., 2016; Yang et al., 2016; Edger et al., 2017; Ramírez‐González et al., 2018; Oberprieler et al., 2019; Landis et al., 2020; Song et al., 2020), altered epigenetic regulation (Madlung et al., 2002; Salmon et al., 2005; Shcherban et al., 2008; Fulneček et al., 2009; Akagi et al., 2016; Chen et al., 2017; Song et al., 2017; Ding and Chen, 2018; Rao et al., 2023), transposable element expansion (Ågren et al., 2016; Baduel et al., 2019), altered rates of homologous, ectopic, and intergenomic recombination (Chalhoub et al., 2014; Guo et al., 2014; Jarvis et al., 2017; Chen et al., 2018; Bertioli et al., 2019; Mason and Wendel, 2020; Gonzalo et al., 2023), chromosomal structural changes (Chester et al., 2012; Edwards et al., 2017; Gordon et al., 2020; Cai et al., 2021; Orantes‐Bonilla et al., 2022), among a host of other fundamental changes to genome biology (Otto, 2007; Leitch and Leitch, 2008; Doyle and Coate, 2019; Bomblies, 2020), all of which have the potential to directly impact organismal function and fitness.

Polyploids are generally categorized into one of two categories: allopolyploid or autopolyploid (Kihara and Ono, 1926; Soltis et al., 2014), depending upon whether their formation occurred via hybridization (Doyle et al., 2008) or via unreduced gametes (Mason and Pires, 2015). The mechanism of formation has important implications for post‐WGD evolution, namely in the pattern of chromosome pairing during meiosis (Comai, 2005). Autopolyploids often exhibit multisomic inheritance in which homologs pair randomly, or even form tetravalents, during meiosis (Ramsey and Schemske, 2003). These unusual meiotic patterns almost certainly explain in part why so many polyploids also reproduce asexually (Otto and Whitton, 2000; Bomblies et al., 2016). By contrast, allopolyploids exhibit a range of different inheritance patterns ranging from completely disomic, in which homoeologs pair with their correct homolog during meiosis (e.g., cotton; Endrizzi, 1962), to tetrasomic, in which homoeologs can pair with the correct homolog or with the homoeolog from the opposing subgenome (e.g., peanut; Leal‐Bertioli et al., 2018). The range of inheritance patterns exhibited by allopolyploid taxa can even be observed within the same genome (e.g., tobacco; Edwards et al., 2017). Pairing and recombination between homoeologs from opposite subgenomes can even result in homoeologous exchange (crossover) or homoeologous gene conversion (non‐crossover), leading to gene flow across subgenomes (Mason and Wendel, 2020), and this process is expected to be especially prominent in relatively young allopolyploids (e.g., peanut, coffee). This homoeologous gene flow (HGF) therefore provides a mechanism for the production of novel genotypes that were absent from the constituent subgenomes at the time of polyploid formation. With the rise of long‐read sequencing and high‐quality polyploid genomic resources, patterns of HGF and some of its phenotypic effects have been documented in a number of allopolyploid systems (Gaeta et al., 2007; Lashermes et al., 2016; Xiong et al., 2020; Zhang et al., 2020; Chu et al., 2021), although the contributions of variation derived from HGF to allopolyploid lineage success remain poorly understood (Deb et al., 2023).

As with inter‐species introgression, HGF may provide allopolyploids access to adaptive variation that has already been tested by nature. Adaptive variation obtained by HGF may be particularly important for maintaining epistatic interactions in the face of genome mergers, as it provides a means to overcome Bateson–Dobzhansky–Muller incompatibilities (BDMIs) that can accompany genome merger (Sharbrough et al., 2017). That is, if HGF can facilitate replacement of incompatible components of epistatic interactions with compatible ones, allopolyploid lineages may be able to avoid some of the deleterious consequences of hybridization. One particularly useful example can be found in the interactions between nuclear‐encoded and cytoplasmically encoded (i.e., mitochondria and chloroplast) genes and gene products, which are critical for carrying out the essential processes of respiration and photosynthesis (Rand et al., 2004; Sloan et al., 2018). In particular, multi‐subunit enzyme complexes that are jointly encoded by the nuclear genome and the cytoplasmic genomes (e.g., Complexes I, III, IV, and V of the electron transport chain in mitochondria, and Rubisco, photosystem I and II, and the Clp protease in chloroplasts; Forsythe et al., 2019) produce the vast majority of the cell's energy budget. Because the cytoplasmic genomes are usually inherited from only a single progenitor (Camus et al., 2022), while nuclear genomes are biparentally inherited from both progenitors, co‐evolution between nuclear and cytoplasmic genes (i.e., cytonuclear co‐evolution) in the maternal lineage can result in incompatibilities between the cytoplasmic genomes and the paternally derived half of the nuclear genome (Sharbrough et al., 2017). Maternally biased HGF provides an opportunity to ameliorate such cytonuclear incompatibilities in allopolyploids (just as co‐introgression can act to maintain epistatic interactions in inter‐species gene flow; Beck et al., 2015), while other epistatic modules may benefit from HGF in either direction.

*Coffea arabica* L. (4*x* = 2*n* = 44) is an economically important allotetraploid crop contributing to ≥65% of global coffee consumption (~170 million bags/year in total; https://www.ico.org/es/Market-Report-22-23-c.asp [accessed August 2023]) (Campuzano‐Duque et al., 2021), and is also an excellent model for studying HGF. In particular, *C. arabica* is the result of a hybridization event between *C. canephora* Pierre ex A. Froehner (2*x* = 2*n* = 22; paternal diploid progenitor) and *C. eugenioides* S. Moore (2*x* = 2*n* = 22; maternal/cytoplasmic diploid progenitor) (Figure 1), with some debate over whether it evolved recently (i.e., ~10,000–50,000 years ago (Cros et al., 1998; Lashermes et al., 1999; Scalabrin et al., 2020) or more anciently (610,000 years ago; Salojarvi et al., 2023). Regardless, there has been sufficient time for HGF to occur, yet the maternal (E subgenome) and paternal (C subgenome) subgenomes remain distinguishable (mean *d*_*S*_ ~ 2.6%, mean *d*_*N*_ ~ 1.0% between diploids; Sharbrough et al., 2022).

**Figure 1:**
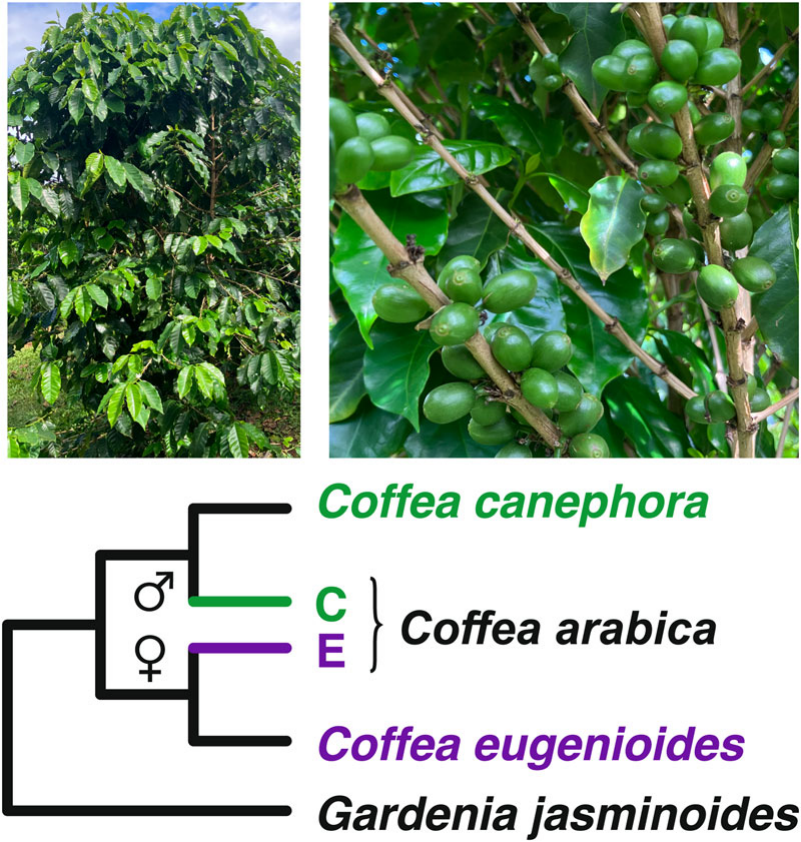
*Coffea arabica* is an allotetraploid formed via hybridization between *C. eugenioides* and *C. canephora*. Top: Images of *C. arabica* in a field in Oahu, Hawaii, USA. Photos by A. Ortiz. Bottom: Cladogram depicting the relationships between *C. eugenioides*, *C. canephora*, hybrid tetraploid *C. arabica*, and outgroup *Gardenia jasminoides*. *Coffea eugenioides* served as the maternal (cytoplasmic) donor in the hybridization event, providing half of the nuclear genome and both organellar genomes (purple line), while *C. canephora* provided the paternal half of the nuclear genome (green line).

Here, we developed a new implementation of the classic ABBA‐BABA test for inter‐species introgression (Durand et al., 2011) to evaluate the direction and extent of HGF in allopolyploid genomes, and we employed it to characterize genome‐wide patterns of HGF in *C. arabica*. To test whether HGF can contribute to the amelioration of epistatic incompatibilities in allopolyploids, we also evaluated whether genes whose products are targeted to the mitochondria and to the chloroplasts are especially likely to experience HGF from the maternally derived subgenome into the paternally derived subgenome, compared to genes whose products are targeted elsewhere in the cell. Overall, we found clear evidence of bi‐directional HGF in the *C. arabica* genome and evidence of maternally biased HGF in nuclear‐encoded genes that function in plastid–nuclear enzyme complexes, but a dearth of HGF (in either direction) in nuclear‐encoded genes whose products are subunits of mitonuclear enzyme complexes. We also compared our method to a tree‐based approach and found very similar patterns across both methods, indicating that the easier‐to‐implement *D*‐statistic approach can be powerfully leveraged for detecting HGF in allopolyploids.

## METHODS

### Developing an ABBA‐BABA test for homoeologous gene flow

A rooted, four‐taxon bifurcating tree can have three possible topologies: (1) (((Sp1, Sp2), Sp3), O), (2) (((Sp1, Sp3), Sp2), O), and (3) (((Sp2, Sp3), Sp1), O), where O represents the outgroup. All three gene tree topologies will be observed if enough genes are sampled from the genome. In the absence of hybridization, the most common gene tree topology among these three possibilities will be identical to the species branching order, while the two rarer gene tree topologies are expected to be approximately equal in abundance, owing to the random fixation of polymorphic alleles across the two sequential splits (i.e., incomplete lineage sorting [ILS]). Overabundance of one ILS tree compared to the other is a robust indicator of hybridization (Forsythe et al., 2020). The traditional ABBA‐BABA test (Durand et al., 2011) takes advantage of this expectation to test whether nucleotide site patterns exhibit asymmetrical abundance across the genome (Appendix S1). Assuming the true species tree of four species can be denoted as (((Sp1, Sp2), Sp3), O), the nucleotide site pattern in which the outgroup and Sp3 share an *ancestral* allele (A), whereas Sp1 and Sp2 share a *derived* allele (B), is consistent with the species branching order. By contrast, a site pattern in which Sp1 and the outgroup share the ancestral allele (A) and Sp2 and Sp3 share the derived allele (B) represents one ILS tree topology (denoted an ABBA site pattern), and the opposite site pattern in which the ancestral allele is shared by Sp2 and the outgroup and the derived allele is shared by Sp1 and Sp3 represents the other ILS tree topology (BABA site pattern). A relatively equal number of discordant ABBA and BABA site patterns is expected under ILS; however, an excess of either the ABBA or the BABA site pattern cannot be explained by ILS alone and requires some form of introgression between Sp2–Sp3 (overabundance of ABBA) or between Sp1–Sp3 (overabundance of BABA).

**Figure 2:**
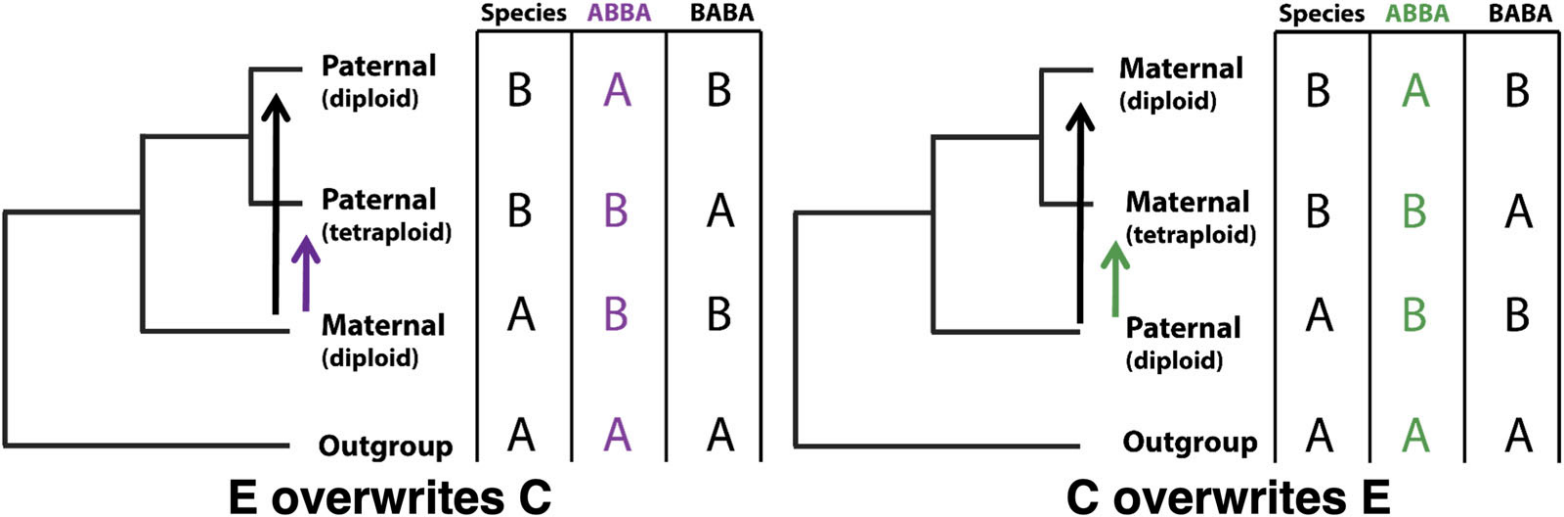
Two‐way, reciprocal ABBA‐BABA test on *Coffea arabica* subgenomes. The two‐way reciprocal ABBA‐BABA test leverages the sister relationship between each subgenome and its corresponding diploid to estimate the rate of homoeologous gene flow (HGF) from the other subgenome (left: maternal overwrites paternal, right: paternal overwrites maternal). The “donor” subgenome is excluded from the test, such that HGF between subgenomes is expected to produce an “ABBA” site pattern, with subgenomes appearing to be more closely related to the opposite diploid than to the diploid from which they were originally derived. The opposite “BABA” site pattern is therefore used as an estimate of the expected frequency of this pattern evolving by random chance (i.e., incomplete lineage sorting [ILS]).

We extended this same logic to test whether HGF has impacted allopolyploid genomes. Specifically, we performed the ABBA‐BABA test in two separate comparisons (Figure 2): (1) testing for HGF from the E genome into the C genome (*D*_*MAT*_), and (2) testing for HGF from the C genome into the E genome (*D*_*PAT*_). For each test, we quantified the number of sites for which derived alleles were shared by one subgenome and the opposing diploid genome (ABBA sites) compared to the number of sites for which derived alleles were shared by the two diploid genomes (BABA sites), using *Gardenia jasminoides* J. Ellis as an outgroup. If the number of ABBA sites was greater than the number of BABA sites (i.e., *D*_*MAT*_ > 0 or *D*_*PAT*_ > 0), we could infer HGF in that particular direction. In essence, this method tests whether the paternally derived subgenome has become more “maternal‐like,” and whether the maternally derived subgenome has become more “paternal‐like,” since the allopolyploidization event. We quantified *D*‐statistics for concatenated alignments of 6672 genes, using 10,000 gene‐level bootstrap replicates to assess statistical significance (Python scripts are available at https://github.com/albuquerque-turkey/Coffea_HGF; see Data Availability Statement). We calculated 95% confidence intervals (CIs) from the gene‐level bootstrap replicates and performed a *Z*‐test to determine whether *D*‐statistics departed significantly from 0. *D*‐statistic point estimates, bootstrap distributions, and 95% CIs were plotted in R v4.1.2 (R Core Team, 2023) using *ggplot2* (Wickham, 2011).

### Orthologous gene alignments and CyMIRA gene classification

Our full HGF analysis was run on 6672 orthologous single‐copy genes (Appendix S2) originally identified in Sharbrough et al. (2022), with alignments available at doi.org/10.6084/m9.figshare.24085830. Briefly, we obtained coding sequences (CDS) from publicly available assemblies for *C. arabica* (GCF_003713225.1), *C. eugenioides* (GCF_003713205.1), *C. canephora* (GCA_900059795.1), and *G. jasminoides* (GCA_013103745.1), and then aligned all 6672 orthologous gene groups with MAFFT v7.480 (Katoh and Standley, 2013), using a Perl wrapper (https://github.com/dbsloan/perl_modules/blob/master/sloan.pm) to convert the CDS to amino acid sequences, align with MAFFT, and then convert the sequences back to nucleotides, as in Sharbrough et al. (2022). We used two distinct alignment trimming strategies: Gblocks v0.91b with the ‘‐n’ parameter set (Castresana, 2000), and ClipKIT v1.2.0 (Steenwyk et al., 2020) with the ‐l parameter set and using a custom Python wrapper to convert ClipKIT‐trimmed amino acid alignments back to CDS alignments (https://github.com/jsharbrough/protTrim2CDS). Gblocks‐trimmed and ClipKIT‐trimmed alignments that were ≥99 bp in length were fed into ABBA‐BABA tests to ensure that the trimming method did not affect our inferences of HGF. In general, Gblocks (Appendix S3) resulted in more stringently trimmed alignments than the ClipKIT‐trimmed alignments.

To evaluate whether HGF was especially maternally biased in genes targeted to, and interacting with, mitochondrial and chloroplast genes and gene products, we used the classifications from Sharbrough et al. (2022), available at https://github.com/jsharbrough/CyMIRA_gene_classification/tree/master/Species_CyMIRA, to classify genes into six categories: non‐organelle‐targeted (NOT); mitochondrial‐targeted, non‐interacting (MTNI); mitochondrial‐targeted, interacting (MTI); mitochondrial enzyme complexes (MTEC); plastid‐targeted, non‐interacting (PTNI); plastid‐targeted, interacting (PTI); and plastid enzyme complexes (PTEC) (Table 1). Of those, the non‐interacting and interacting categories were mutually exclusive for each organelle, while genes involved in enzyme complexes were a subset of interacting genes that are involved in cytonuclear enzyme complexes, as defined by CyMIRA v1.0 (Forsythe et al., 2019). Mitochondrial enzyme complexes included the mitochondrial twin arginine translocation (TAT) complex, the mitochondrial ribosome, NADH dehydrogenase (Complex I of the oxidative phosphorylation cascade [OXPHOS]), ubiquinol–cytochrome c reductase (Complex III of OXPHOS), cytochrome c oxidase (Complex IV of OXPHOS), and ATP synthase (Complex V of OXPHOS). Plastid enzyme complexes included the heteromeric acetyl‐CoA carboxylase (ACCase), the chloroplast ribosome, the Clp protease, both photosystems I and II, Rubisco, the chloroplast NADH dehydrogenase‐like complex (NDH), cytochrome b6f, and the chloroplast ATP synthase. Gene names for each of these complexes from all five species are available in Appendix S4.

**Table 1:** Functional classification of single‐copy orthologous genes in *Coffea*.

Category	No. of genes	Category description
All	6672	Genome‐wide set of single‐copy orthologous genes
NOT	5581	Genes that are not targeted to the mitochondria or chloroplasts
MTNI	536	Genes whose products are targeted to the mitochondria but do not interact with mitochondrial genes or gene products
MTI	204	Genes whose products are targeted to the mitochondria and interact with mitochondrial genes or gene products
MTEC	59	Subset of MTI; genes whose products are involved in mitonuclear enzyme complexes
PTNI	731	Genes whose products are targeted to the chloroplasts but do not interact with plastid genes or gene products
PTI	140	Genes whose products are targeted to the chloroplasts and interact with plastid genes or gene products
PTEC	48	Subset of PTI; genes whose products are involved in plastid–nuclear enzyme complexes

#### Phylogenetic analyses

To validate the inferences made by our newly developed method, we inferred phylogenetic trees by maximum likelihood (ML) using RAxML v8.2.12 (Stamatakis, 2014). For each gene tree, we used the raxmlHPC‐PTHREADS function, employing the rapid bootstrap analysis and search for best‐scoring ML tree in one program run (‘‐f a’ parameter), with 100 bootstrap replicates, and assuming the GTRGAMMAIX model of molecular evolution. We rooted each of the resulting gene trees with the *G. jasminoides* sequence and determined which of the 15 possible gene tree topologies (Appendix S5) each gene tree fit. We reasoned that if HGF was unidirectional in a given gene, tetraploids would be expected to be sister to each other, with the direction of HGF matching the most closely related diploid (tree topology L or M; Appendix S6). Of course, these topological patterns can also be produced by ILS, recurrent mutations, and autapomorphies; however, in the absence of HGF, the probability of the tetraploids to be sister to each other should be equal to the probability of a tetraploid being sister to the opposing diploid (tree topology N or H) and to that of the two diploids being sister to one another (tree topology I or O). Similarly, if HGF resulted in a reciprocal exchange of DNA, we would observe a tree in which tetraploid sequences would group with the opposing subgenome (tree topology F), while trees derived from random processes would find the diploids as sister to one another and the tetraploids as sister to one another (tree topology K). The relative abundance of the HGF trees compared to their ILS‐derived alternatives provides a similar comparison to the *D*‐statistic, but based on whole genes and an explicit model of molecular evolution. We compared HGF tree topology abundance using a series of binomial, χ^2^, and Fisher's exact tests, correcting for multiple comparisons using the Holm procedure for the Bonferroni correction (Holm, 1979).

We also tested whether putative HGF trees were especially abundant among mitochondrial and plastid enzyme complex genes, using a Fisher's exact test to determine whether cytonuclear enzyme complex genes exhibited an increase in the ratio of maternally biased HGF trees compared to non‐organelle‐targeted genes.

## RESULTS

### Testing for HGF in allotetraploid *Coffea arabica*

**Figure 3:**
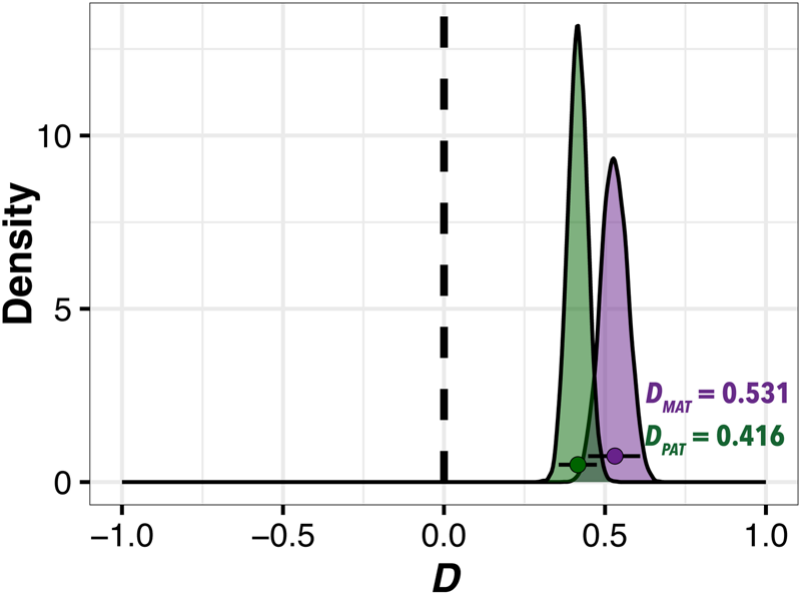
Genome‐wide patterns of bidirectional HGF in allotetraploid *Coffea arabica*. Reciprocal *D*‐statistic estimates of the E subgenome overwriting the C subgenome (purple, *D*_*MAT*_) and of the C subgenome overwriting the E subgenome (green, *D*_*PAT*_). Points represent overall *D*‐statistics, density plots depict distributions from 10,000 gene‐level bootstrap replicates, and error bars represent 95% CIs. Distributions that are significantly greater than 0 are indicative of HGF in that direction.

We used a stepwise, reciprocal ABBA‐BABA framework to evaluate whether homoeologous exchange and homoeologous gene conversion contribute to gene flow between subgenomes of allotetraploid *C. arabica*. These analyses of the ClipKIT‐trimmed alignments of 6672 orthologous genes revealed strong evidence of bidirectional HGF (i.e., *D*_*MAT*_ and *D*_*PAT*_ >> 0; Table 2), as we saw extensive overwriting of the C subgenome by the E subgenome (maternally derived HGF) and overwriting of the E subgenome by the C subgenome (paternally derived HGF) (Figure 3). Qualitatively similar results were obtained from Gblocks‐trimmed alignments, which are provided in Appendices S3 and S7, and none of the gene categories showed different statistical patterns based on trimming method. Maternally derived HGF (*D*_*MAT*_ [CI_95_] = 0.538 [0.448–0.610]) was slightly, but not significantly, greater than paternally derived HGF after correcting for multiple comparisons (*D*_*PAT*_ [CI_95_] = 0.416 [0.357–0.475]; *Z* = 2.785; *P* = 0.0026). Overall, our new method indicated the presence of substantial and bidirectional HGF in *C. arabica* genomes.

**Table 2:** Reciprocal ABBA‐BABA test statistics across gene functional categories.

Category^a^	*D*_*MAT*_ (CI_95_ b)	*Z*‐score	*P* c	*D*_*PAT*_ (CI_95_ b)	*Z*‐score	*P* c
All	0.514 (0.448, 0.609)	12.908	<0.0001*	0.416 (0.356, 0.475)	13.749	<0.0001*
MTNI	0.572 (0.268, 0.812)	4.384	<0.0001*	0.022 (−0.343, 0.417)	2.375	0.0088
MTI	0.318 (−0.153, 0.716)	3.27	0.0005*	0.450 (−0.288, 0.846)	4.466	<0.0001*
MTEC	−0.704 (−0.903, −0.133)	−0.965	0.1672	−0.600 (−0.832, 0.615)	1.464	0.0715
PTNI	0.493 (0.257, 0.705)	5.718	<0.0001*	0.134 (−0.398, 0.562)	3.635	0.0001*
PTI	0.520 (0.144, 0.839)	8.313	<0.0001*	−0.222 (−0.598, 0.516)	7.036	<0.0001*
PTEC	0.888 (0.378, 0.987)	5.499	<0.0001*	−0.581 (−0.921, 0.839)	1.522	0.0641

^a^ See Table 1 for gene category descriptions.

^b^ 95% Confidence intervals were produced using 10,000 gene‐level bootstrap replicates.

^c^ *P* values reflect comparison to absence of homoeologous gene flow (*D* = 0).

* Significant after correcting for multiple comparisons. Additional pairwise comparisons made using ClipKIT‐trimmed data can be found in Appendix S9. Results from Gblocks‐trimmed data can be found in Appendix S7.

**Figure 4:**
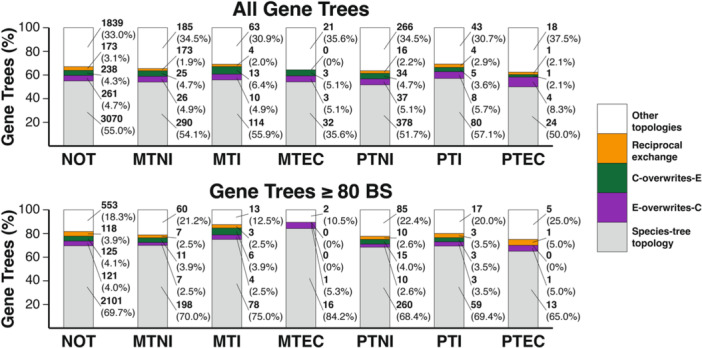
Single‐copy orthologous gene tree topologies in *Coffea*. Percentage of gene trees (top: all trees, bottom: only trees with >80% bootstrap support) of various tree topologies (gray = species topology; purple = E‐overwriting‐C; green = C‐overwriting‐E; orange = reciprocal exchange; white = other) in single‐copy orthologous gene groups.

To evaluate the robustness of these results, we also inferred phylogenetic trees for each of these 6672 genes. Overall, the majority of genes exhibited the same topology as the species tree (3671/6672, 54.7%); however, a sizeable fraction of genes exhibited alternative topologies, and all 15 possible tree topologies were observed in these genes (Table 3, Figure 4). Among these, there were 1115 genes (16.7%) in which the E subgenome, the C subgenome, and *C. eugenioides* formed a clade to the exclusion of *C. canephora*. Of these, 316 genes (4.7%) exhibited sister relationships between the E and C subgenomes. We denote these 316 trees as putative E‐overwriting‐C trees. Similarly, there were 1221 genes (18.3%) in which the C subgenome, the E subgenome, and *C. canephora* all formed a clade to the exclusion of *C. eugenioides*, with 289 genes (4.3%) in which the subgenomes were sister to one another (putative C‐overwriting‐E trees). There were also 198 genes (3.0%) in which the C subgenome grouped with *C. eugenioides* and the E subgenome grouped with *C. canephora* (putative reciprocal exchange trees). Together, these phylogenetic results provide a very similar picture as the reciprocal ABBA‐BABA test, with a substantial fraction of the *C. arabica* genome exhibiting patterns consistent with HGF.

**Table 3:** Distribution of gene tree topologies across functional gene categories.^a^

Topology^b^	ALL	NOT	MTNI	MTI	MTEC	PTNI	PTI	PTEC
A^c^	3651	3070	290	114	32	378	80	24
B	478	410	30	10	1	49	8	1
C	278	230	24	8	7	36	4	2
D	474	396	39	12	2	55	9	5
E	629	507	67	18	4	88	11	3
F^d^	198	173	10	4	0	16	4	1
G	89	75	6	2	2	8	4	3
H	34	31	2	0	0	3	0	0
I	19	13	2	2	0	4	0	0
J	68	57	6	2	0	7	3	1
K	81	63	8	5	1	11	2	1
L^e^	316	261	26	10	3	37	8	4
M^f^	289	238	25	13	3	34	5	1
N	37	31	0	2	2	2	2	2
O	31	26	1	2	2	3	0	0
**Total**	**6672**	**5581**	**536**	**204**	**59**	**731**	**140**	**48**

^a^ See Table 1 for gene category descriptions.

^b^ See Appendix S5 for all gene tree topologies.

^c^ Species tree.

^d^ Tree consistent with reciprocal exchange across subgenomes.

^e^ Tree consistent with C subgenome overwriting E subgenome.

^f^ Tree consistent with E subgenome overwriting C subgenome.

To evaluate the likelihood that these trees arose through ILS, we compared the number of putative E‐overwriting‐C trees, the number of putative C‐overwriting‐E trees, and the number of putative reciprocal exchange trees to the number of other trees that would be expected by ILS (Appendix S6). We found significantly higher numbers in all three sets of putative HGF gene trees compared to their ILS counterparts (Appendix S8), a pattern that was true even when restricting the analysis to those trees with ≥80 bootstrap support. Together, the prevalence of putative HGF trees is unlikely to be explained entirely by ILS, indicating that HGF has played a prominent role in the genealogical history of the *C. arabica* genome.

### Maternally biased HGF in plastid‐targeted, but not mitochondrial‐targeted nuclear genes

**Figure 5:**
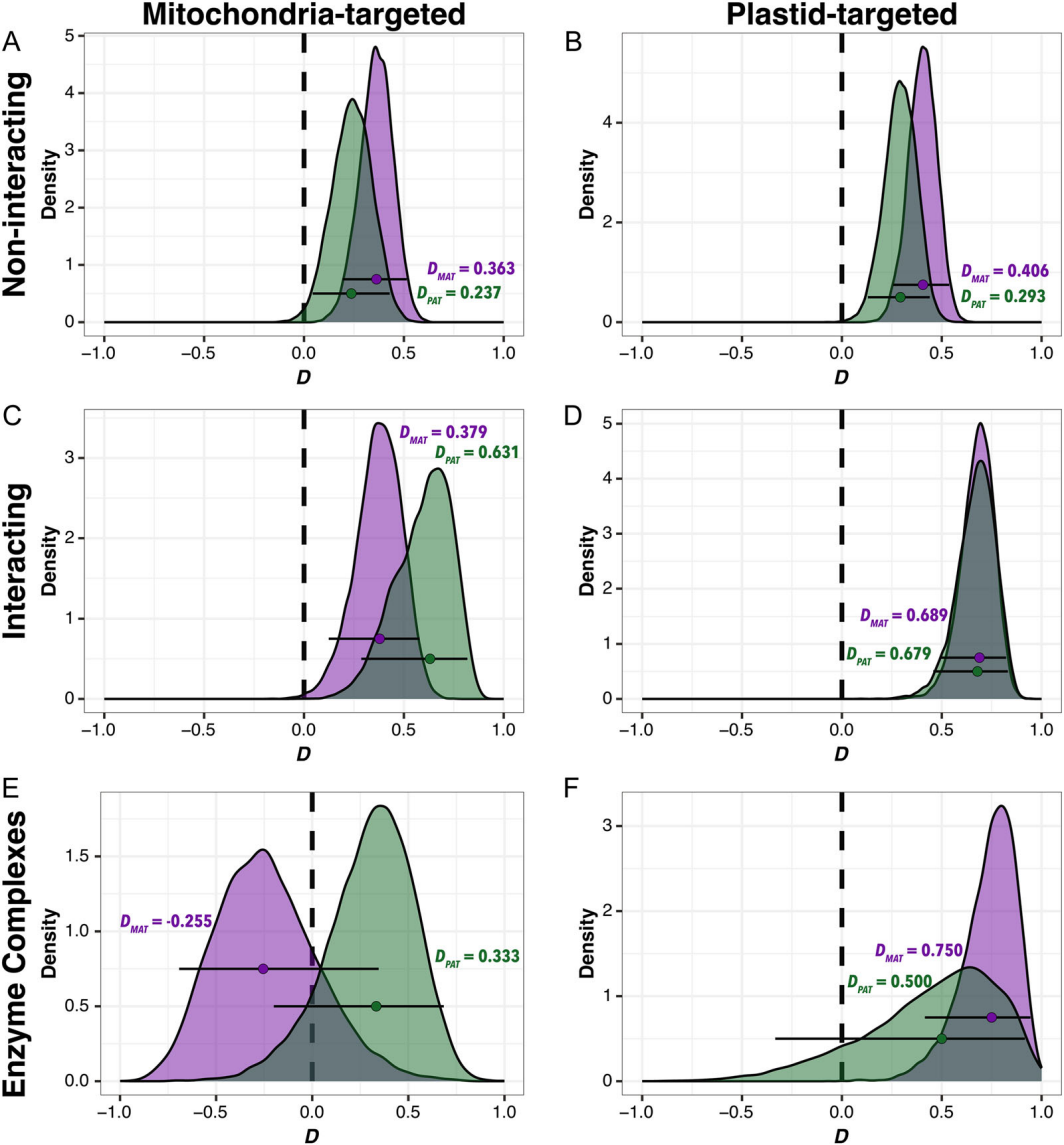
Patterns of HGF in mitochondria‐ and plastid‐targeted genes in allotetraploid *Coffea arabica*. Reciprocal *D*‐statistic estimates of the E subgenome overwriting the C subgenome (purple, *D*_*MAT*_) and of the C subgenome overwriting the E subgenome (green, *D*_*PAT*_) in genes targeted to the mitochondria (left) and to the plastid (right). Genes are grouped according to the intimacy of interaction: (A, B) non‐interacting, (C, D) interacting, (E, F) enzyme complexes. Points represent overall *D*‐statistics, density plots depict distributions from 10,000 gene‐level bootstrap replicates, and error bars represent 95% CIs. Distributions that are significantly greater than 0 are indicative of HGF in that direction.

*Coffea arabica* received both its mitochondrial and plastid genomes from *C. eugenioides*, but nuclear DNA from both *C. eugenioides* and *C. canephora* (Cros et al., 1998). Because interactions between nuclear‐encoded genes and mitochondrial‐ and plastid‐encoded genes are critical for plant function and fitness (Kremnev and Strand, 2014; Kühn et al., 2015), mutational changes in one genome or the other are expected to produce intense selection for compensatory changes to maintain respiratory and photosynthetic function (Rand et al., 2004). It therefore stands to reason that co‐adapted mitonuclear and plastid–nuclear epistatic interactions might be disrupted upon genome merger (Sharbrough et al., 2017). If conflict between the paternally derived nuclear subgenome and the maternally derived cytoplasmic genomes exists, HGF provides a rapid evolutionary mechanism to ameliorate the cytonuclear mismatch by replacing paternally derived genes targeted to the energy‐producing organelles with maternally derived genes. We tested this hypothesis using our reciprocal ABBA‐BABA method in sets of organelle‐targeted non‐interacting genes, interacting genes, and genes involved in cytonuclear enzyme complexes. There was no apparent difference between genome‐wide patterns of HGF and organelle‐targeted genes that do not interact with organelle genes or gene products (*n*_MTNI_ = 536; *n*_PTNI_ = 731; Figures 5A, 5B). Interacting genes (*n*_MTI_ = 204; *n*_PTI_ = 140) also showed a similar pattern (Figures 5C, 5D), but we found substantially different patterns of HGF compared to non‐organelle‐targeted genes in the subset of interacting genes that are involved in cytonuclear enzyme complexes (*n*_MTEC_ = 59; *n*_PTEC_ = 48). Mitochondrial enzyme complex genes exhibited no evidence of HGF in either direction (*D*_*MAT*_ [CI_95_] = −0.255 [−0.692, 0.345]; *Z* = −0.965; *P* = 0.167; *D*_*PAT*_ [CI_95_] = 0.333 [−0.200, 0.684]; *Z* = 1.464; *P* = 0.0715) (Figure 5E), indicating that HGF is not acting to ameliorate mitonuclear conflicts in *C. arabica*. By contrast, plastid enzyme complex genes exhibited substantial amounts of maternally biased HGF (*D*_*MAT*_ [CI_95_] = 0.750 [0.415, 0.944]; *Z* = 5.499; *P* < 0.0001), but no evidence of paternally biased HGF (*D*_*PAT*_ [CI_95_] = 0.500 [−0.333, 0.916]; *Z* = 1.522; *P* = 0.0641) (Figure 5F, Appendix S9). This pattern of the E subgenome overwriting the C subgenome in this set of genes is consistent with a scenario in which cytonuclear mismatches caused by the paternally derived nuclear subgenome are ameliorated by maternally biased HGF.

**Figure 6:**
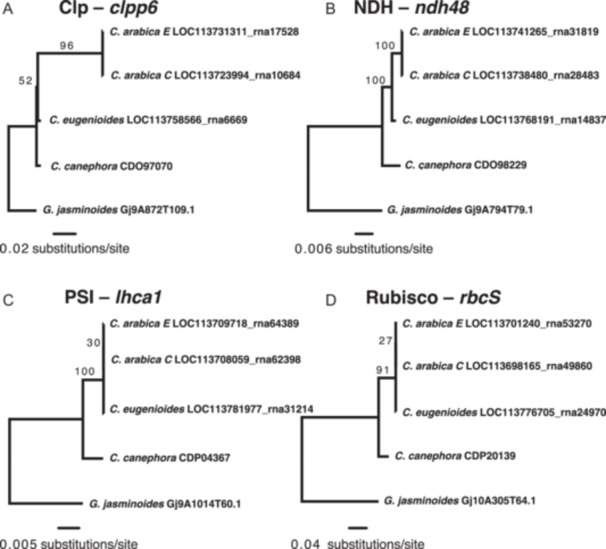
Gene trees of four plastid enzyme complex genes that appear to exhibit maternally biased HGF. (A) Gene tree for *clpp6*, a nuclear‐encoded gene whose protein product participates in the heteromeric ring of the Clp protease. (B) Gene tree for *ndh48*, a nuclear‐encoded gene whose protein product is involved in the plastid NDH. (C) Gene tree for *lcha1*, a nuclear‐encoded gene whose protein product is involved in photosystem I (PSI). (D) Gene tree for *rbcS*, a nuclear‐encoded gene whose protein product represents the small subunit of the Rubisco protein complex.

We also tested whether cytonuclear enzyme complex genes exhibited evidence of maternally biased HGF based on gene trees. We observed that a higher proportion of plastid enzyme complex genes exhibited the E‐overwriting‐C topology (*n* = 4, 8.3%) and a lower proportion of plastid enzyme complex genes exhibited the C‐overwriting‐E topology (*n* = 1, 2.1%) compared to the non‐organelle‐targeted genes (*n*_E‐over‐C_ = 261, 4.7%; *n*_C‐over‐E_ = 238, 4.3%), but neither difference was significant (Fisher's exact test, *P*_E‐over‐C_ = 0.274; *P*_C‐over‐E_ = 1.0). Notably, the genes that did exhibit maternally biased HGF topologies are all central members of four distinct and essential plastid enzyme complexes: Rubisco, NDH, photosystem I, and Clp (Figure 6). An equal number of mitochondrial enzyme complex genes exhibited the E‐overwriting‐C topology as the C‐overwriting‐E topology (*n* = 3, 5.1% for each), which were not different from the pattern in non‐organelle‐targeted genes (*P*_E‐over‐C_ = 0.752; *P*_C‐over‐E_ = 0.737). Although the patterns of HGF in plastid‐targeted enzyme complex genes were not significantly different from the genome‐wide pattern, this change in proportion is in the direction we predicted (and is consistent with our reciprocal ABBA‐BABA results), potentially indicating that the phylogenetic approach lacks the power to reveal biased HGF as effectively as our newly implemented method.

## DISCUSSION

### A stepwise, reciprocal ABBA‐BABA test to detect HGF in allopolyploid genomes

Recombination between homoeologs provides an important source of novel variation for allopolyploids (Mason and Wendel, 2020), and characterizing patterns and consequences of HGF represents an important endeavor for understanding evolutionary dynamics after WGDs (Deb et al., 2023). This can be done in a number of ways (Schiessl et al., 2019) including comparative read mapping with long reads (e.g., Jarvis et al., 2017; Chu et al., 2021; Oruganti et al., 2023), single‐nucleotide (or more) variant comparisons (e.g., Salmon et al., 2010; Lashermes et al., 2016; Conover et al., 2023), structural variant characterization (e.g., Orantes‐Bonilla et al., 2022), and even cytogenetic tools like fluorescent in situ hybridization (e.g., Stein et al., 2017) or optical mapping (e.g., Yuan et al., 2018). The method we implemented here incorporates the logic developed by the introgression literature (recently reviewed by Hibbins and Hahn, 2022) to evaluate HGF against an ILS framework. The ABBA‐BABA statistic has been used previously to test for the presence of interspecies introgression in polyploid genomes (e.g., Pont et al., 2019), but here we repurposed it to characterize patterns of introgression *within the same cell*.

Our method can also look for patterns of HGF in functionally related genes (e.g., genes whose products are targeted to the mitochondria and plastids). Because these genes are dispersed across the genome and not closely linked, it is possible to infer the activity of natural selection acting to fix HGF in a particular direction. Our current implementation is powerful enough to detect (or not detect) HGF with as few as ~50 genes, but smaller gene sets could likely be tested by bootstrapping at the site level, rather than at the gene level. Relatedly, a sliding window approach may be able to identify regions of HGF, as is implemented in the ${\hat{f}}_{d}$ statistic (Martin et al., 2014). Because regions of HGF caused by homoeologous gene conversion are expected to span ≤200 bp, even single‐gene phylogenetic analyses of HGF may miss biologically important events. Moreover, such a sliding window approach will be useful for characterizing the size of genomic blocks that can be produced by neutral processes like ILS and, as a result, will define our baseline for HGF at the local scale. In this way, we could potentially account for two limitations of the *D*‐statistic: (1) artifactual inflation of *D* due to small numbers of loci being included, and (2) accounting for artifactual inflation of *D* caused by low effective population size, which could be estimated by the background ILS level.

Importantly, this reciprocal ABBA‐BABA approach requires that introgression between diploid relatives not be extensive, as that would increase the abundance of BABA sites in our test and obscure patterns of HGF. Therefore, we recommend testing for diploid introgression prior to testing for HGF (we found no evidence of introgression between *C. eugenioides* and *C. canephora* here). Additional important caveats of the ABBA‐BABA approach are that it assumes the comparison of similar (and relatively large) effective population sizes across the taxa, as well as similar rates of evolution across genomes. For example, Frankel and Ané (2023) found high false positive rates in tests of introgression (e.g., *D*_3_, ABBA‐BABA, and HyDe) pertaining to lineages with differing rates of evolution. However, the ABBA‐BABA test remained at least somewhat robust to differential rates of lineage evolution as compared to the three‐taxon *D*_3_ test, which was most sensitive to such variation. In this case, the polyploid subgenomes likely have a reduced effective population size compared to their diploid progenitors as a result of the post‐WGD bottleneck that appears to have occurred in *C. arabica* (Mekbib et al., 2022). However, this bottleneck applied to both subgenomes, meaning that they are expected to be similarly affected. Similarly, rates of allopolyploid subgenome evolution have been found to differ from one another (Sharbrough et al., 2022), and differ systematically from their diploid progenitors (e.g., Conover and Wendel, 2022), meaning that some false positive inferences of HGF from this method (particularly on small regions or small gene sets) could be possible. Importantly, however, the test does not rely on direct comparisons between the subgenomes, thus subgenomic differences in relative evolutionary rate should not impact the inference of HGF.

Our approach also relies upon high‐quality genome assemblies of the polyploid subgenomes, the two diploid relatives, and of an outgroup. Due to the availability of PacBio HiFi reads, Hi‐C (or similar) sequencing, and ultra‐long Oxford Nanopore reads, among other technologies, such genomic resources are becoming increasingly available. The coffee genomes used in the present study may represent an important source of error, as they contain numerous gaps (the *C. arabica* assembly GCF_003713225.1 comprises 3522 separate contigs), such that misassemblies could contribute to overestimating the rate of HGF. Continuing to improve genome assemblies (and producing pangenomes) will greatly facilitate our ability to evaluate the patterns and consequences of HGF in allopolyploids. Moreover, additional applications of the *D*_3_ test statistic (Hahn and Hibbins, 2019) may allow for robust inferences of HGF in taxa that lack a high‐quality outgroup genome sequence, although we note the caveat above regarding the potential for false positives due to variation in rate across diploid and polyploid genomes.

In our current approach, we only considered single‐copy genes, but expanding beyond these simple cases represents a major area for development in the investigation of polyploid genomes. Indeed, the repeated and cyclical nature of WGDs in plants (Wendel, 2015) means that many, or perhaps even most, genes in plant genomes have paralogs elsewhere in the genome. Importantly, we expect multi‐copy genes to be hotspots for inter‐genomic recombination and homoeologous conversion (Feliner and Rosselló, 2012), just as they are hotspots for intra‐genomic recombination and gene conversion (Brown et al., 1972; Zimmer et al., 1980; Meagher et al., 1989); therefore, developing a framework for identifying rates and patterns of homoeologous recombination in multi‐copy genes will be critical for understanding how allopolyploid genomes evolve post‐WGD (Leitch and Leitch, 2008; Wendel et al., 2016). The phylogenies underlying plant paralogs can also be complex (Clegg et al., 1997; Nei and Rooney, 2005); for example, inter‐copy homogenization has long been documented (Meagher et al., 1989; Clegg et al., 1997; Kovarik et al., 2005), and the redundancy of gene copies makes them likely candidates for lineage‐specific loss. Gong and colleagues investigated homoeologous recombination in several plant polyploids (i.e., *Arachis*, *Arabidopsis*, *Brassica*, *Gossypium*, and *Nicotiana*) in the frequently multi‐copy *rbcS* gene and found a consistent signature of maternally biased homoeologous conversion in each of those species (Gong et al., 2012, 2014). The detailed analysis performed by this group would be challenging to scale to whole genomes (or even to hundreds of genes) because the alignments used in these studies were highly curated and each SNP position was evaluated individually. To foster a genomic perspective, whole‐genome alignment methods like AnchorWave (Song et al., 2022) can enable comparisons in a larger fraction of the genome (Conover et al., 2023), but the gene conversion dynamics of multi‐gene families will largely be missed by such an approach. Including multi‐gene gene family dynamics will require taking advantage of recent advances in gene‐tree–species‐tree reconciliation (e.g., Mirarab et al., 2014; Delabre et al., 2020; Morel et al., 2020; Parey et al., 2020; Willson et al., 2022) to identify duplication and loss events, after which the prospect of identifying homoeologous recombination events would be tractable.

### Patterns of HGF in organelle‐targeted genes

Hybrid incompatibilities between nuclear and cytoplasmic genomes can be produced via cytonuclear co‐evolution, and this special class of BDMIs can play a prominent role in reinforcing species boundaries (Burton and Barreto, 2012; Sloan et al., 2017). Polyploidy may offer a general solution to cytonuclear incompatibilities, as the maternally derived nuclear genes are retained following the genome merger event. As a result, those genes can act as sources of epistatic variation co‐adapted with the cytoplasmic genomes. This is expected to result in either the loss of paternally derived genes encoding products targeted to the mitochondria or chloroplasts, HGF that overwrites paternally derived homoeologs with maternally derived copies, or rapid evolution in paternally derived copies under selection to more closely “match” cytoplasmic interacting partners (Sharbrough et al., 2017).

In a broad sampling of angiosperm allotetraploids, Sharbrough et al. (2022) demonstrated that paternally derived organelle‐targeted genes do not experience global accelerations in rate and are not lost at higher rates than maternally derived copies. A number of allopolyploid angiosperms exhibit evidence of maternally biased homoeologous gene conversion of *rbcS*, the nuclear‐encoded subunit of Rubisco (Gong et al., 2012, 2014; Li et al., 2020). Here, we found that paternally derived genes whose products are involved in jointly encoded plastid–nuclear enzyme complexes are preferentially overwritten by maternally derived genes, but no such pattern was observed in paternally derived genes involved in mitochondrial–nuclear enzyme complexes.

There are several potential explanations for the discrepancy across organellar compartments. First, plastid genomes typically evolve at much higher rates than mitochondrial genomes in angiosperms (Wolfe et al., 1987), and cytonuclear incompatibilities are unlikely to occur in the absence of accelerated rates of cytoplasmic genome evolution (Havird et al., 2015; Rockenbach et al., 2016; Williams et al., 2019). Indeed, the apparent lack of HGF in either direction in mitochondrial enzyme complex genes that we observed here may reflect an absence of mitonuclear incompatibilities in *C. arabica*, owing to a low rate of mitochondrial genome evolution and low sequence divergence between mitochondrial genomes of *C. eugenioides* and *C. canephora*. Second, the efficacy of selection acting on plastid–nuclear interactions may be greater than that acting on mitochondrial–nuclear interactions. Plastid enzyme complexes are expressed at much higher rates than mitochondrial enzyme complexes (Forsythe et al., 2022), to the extent that Rubisco is likely the most abundant enzyme on Earth (Ellis, 1979; Raven, 2013). Genes expressed at higher levels experience more effective selection than genes expressed at lower levels (Drummond et al., 2005; Yang et al., 2012). Additionally, plastid genomes are retained at copy numbers that are orders of magnitude higher than mitochondrial genomes (Fernandes Gyorfy et al., 2021), which is expected to produce a higher effective population size in plastid genomes than mitochondrial genomes.

### Summary and Conclusions

Here, we implemented a repurposing of an existing method for detecting introgression between species into a method for detecting gene flow within a cell and tested this method in the allotetraploid angiosperm *C. arabica*. This proof‐of‐principle for a simple method to detect HGF in allopolyploid taxa provides an important tool for characterizing the impacts of homoeologous exchange and gene conversion in allopolyploids. We also document patterns of maternally biased HGF in plastid‐targeted, but not mitochondria‐targeted, genes that are involved in plastid–nuclear enzyme complexes.

## AUTHOR CONTRIBUTIONS

A.J.O. wrote the code for the method, completed all analyses presented here, and wrote the first draft of the manuscript. J.S. conceived of the method, assisted with validating code, and edited the manuscript. Both authors contributed equally to figure and table development. Both authors approved the final version of the manuscript.

### OPEN RESEARCH BADGES

This article has earned an Open Data badge for making publicly available the digitally shareable data necessary to reproduce the reported results. The data are available at https://doi.org/10.6084/m9.figshare.24085830 and https://github.com/albuquerque-turkey/Coffea_HGF.

## Supporting information


**Appendix S1.** Graphical depiction of the classic implementation of the ABBA‐BABA test. The relative abundance of ABBA (middle tree) vs. BABA (right tree) site patterns in genome‐wide alignments can be used to infer introgression between species in a four‐taxon arrangement.

**Appendix S2.** Orthologous gene groups in *Coffea* species. Orthogroups were inferred after running whole proteomes through OrthoFinder v2, then a combination of phylogenetic (PhyML v3.3) and syntenic (pSONIC v1) information was used to extract single‐copy orthologous gene groups from orthogroups.

**Appendix S3.** Patterns of HGF in genome‐wide and mitochondria‐ and plastid‐targeted genes using Gblocks‐trimmed alignments in allotetraploid *Coffea arabica*. Reciprocal *D*‐statistic estimates from Gblocks‐trimmed alignments of the E subgenome overwriting the C subgenome (purple, *D*_*MAT*_) and of the C subgenome overwriting the E subgenome (green, *D*_*PAT*_) in all genes (top panel) and in genes targeted to the mitochondria (left) and to the plastid (right). Genes are grouped according to the intimacy of interaction: non‐interacting – top panels, interacting – middle panels, enzyme complexes – bottom panels. Points represent overall *D*‐statistics, density plots depict distributions from 10,000 gene‐level bootstrap replicates, and error bars represent 95% CIs. Distributions that are significantly greater than 0 are indicative of HGF in that direction.

**Appendix S4.** CyMIRA cytonuclear enzyme complex gene names in *Coffea*, listing genes whose products are targeted to the mitochondria and chloroplasts and interact with cytoplasmically encoded gene products.

**Appendix S5.** Graphical depiction of the possible gene tree topologies for rooted, five‐taxon trees. There are 15 possible tree topologies for a rooted tree with five taxa. The *Coffea* species tree is depicted in (A) (highlighted in gray). The gene tree that would be expected if HGF were reciprocal across subgenomes is depicted in (F) (highlighted in orange). The gene tree that would be expected if HGF were maternally biased (i.e., E‐overwriting‐C) is depicted in (L) (highlighted in purple). The gene tree that would be expected if HGF were paternally biased (i.e., C‐overwriting‐E) is depicted in (M) (highlighted in green).

**Appendix S6.** Graphical depiction of species tree, HGF trees, and ILS tree topologies, showing the comparison between putative HGF gene trees with alternative gene tree topologies that are due to random sorting of alleles, recurrent mutations, and autapomorphies. See Appendix S5 for the possible gene tree topologies.

**Appendix S7.** Stepwise ABBA‐BABA statistics across gene functional categories for Gblocks‐trimmed alignments.

**Appendix S8.** Tests of prevalence of putative HGF gene trees against random expectations.

**Appendix S9.** Stepwise ABBA‐BABA statistics across gene functional categories for ClipKIT‐trimmed alignments.

## Data Availability

All genomic data used in this paper are publicly available. Alignments and trees for orthologous gene groups are available at https://doi.org/10.6084/m9.figshare.24085830. All code developed for this project is available at https://github.com/albuquerque-turkey/Coffea_HGF. Genomes are available at the National Center for Biotechnology Information (NCBI) under the following accessions: *Coffea arabica* (GCF_003713225.1), *C. eugenioides* (GCF_003713205.1), *C. canephora* (GCA_900059795.1), and *Gardenia jasminoides* (GCA_013103745.1).

## References

[aps311584-bib-0001] Ågren, J. A. , H.‐R. Huang , and S. I. Wright . 2016. Transposable element evolution in the allotetraploid *Capsella bursa‐pastoris* . American Journal of Botany 103: 1197–1202. 27440791 10.3732/ajb.1600103

[aps311584-bib-0002] Akagi, T. , I. M. Henry , T. Kawai , L. Comai , and R. Tao . 2016. Epigenetic regulation of the sex determination gene *MeGI* in polyploid persimmon. Plant Cell 28: 2905–2915. 27956470 10.1105/tpc.16.00532 PMC5240738

[aps311584-bib-0003] Akama, S. , R. Shimizu‐Inatsugi , K. K. Shimizu , and J. Sese . 2014. Genome‐wide quantification of homeolog expression ratio revealed nonstochastic gene regulation in synthetic allopolyploid *Arabidopsis* . Nucleic Acids Research 42: e46. 24423873 10.1093/nar/gkt1376 PMC3973336

[aps311584-bib-0004] Baduel, P. , L. Quadrana , B. Hunter , K. Bomblies , and V. Colot . 2019. Relaxed purifying selection in autopolyploids drives transposable element over‐accumulation which provides variants for local adaptation. Nature Communications 10: 5818. 10.1038/s41467-019-13730-0 PMC6925279 31862875

[aps311584-bib-0005] Beck, E. A. , A. C. Thompson , J. Sharbrough , E. Brud , and A. Llopart . 2015. Gene flow between *Drosophila yakuba* and *Drosophila santomea* in subunit V of cytochrome c oxidase: A potential case of cytonuclear cointrogression. Evolution; International Journal of Organic Evolution 69: 1973–1986. 26155926 10.1111/evo.12718 PMC5042076

[aps311584-bib-0006] Bertioli, D. J. , J. Jenkins , J. Clevenger , O. Dudchenko , D. Gao , G. Seijo , S. C. M. Leal‐Bertioli , et al. 2019. The genome sequence of segmental allotetraploid peanut *Arachis hypogaea* . Nature Genetics 51: 877–884. 31043755 10.1038/s41588-019-0405-z

[aps311584-bib-0007] Bomblies, K. 2020. When everything changes at once: Finding a new normal after genome duplication. Proceedings of the Royal Society B: Biological Sciences 287: 20202154. 10.1098/rspb.2020.2154 PMC7739491 33203329

[aps311584-bib-0008] Bomblies, K. , G. Jones , C. Franklin , D. Zickler , and N. Kleckner . 2016. The challenge of evolving stable polyploidy: Could an increase in ‘crossover interference distance’ play a central role? Chromosoma 125: 287–300. 26753761 10.1007/s00412-015-0571-4 PMC4830878

[aps311584-bib-0009] Brown, D. D. , P. C. Wensink , and E. Jordan . 1972. A comparison of the ribosomal DNA's of *Xenopus laevis* and *Xenopus mulleri*: The evolution of tandem genes. Journal of Molecular Biology 63: 57–73. 5016971 10.1016/0022-2836(72)90521-9

[aps311584-bib-0010] Burton, R. S. , and F. S. Barreto . 2012. A disproportionate role for mtDNA in Dobzhansky‐Muller incompatibilities? Molecular Ecology 21: 4942–4957. 22994153 10.1111/mec.12006

[aps311584-bib-0011] Cai, X. , L. Chang , T. Zhang , H. Chen , L. Zhang , R. Lin , J. Liang , et al. 2021. Impacts of allopolyploidization and structural variation on intraspecific diversification in *Brassica rapa* . Genome Biology 22: 166. 34059118 10.1186/s13059-021-02383-2 PMC8166115

[aps311584-bib-0012] Campuzano‐Duque, L. F. , J. C. Herrera , C. Ged , and M. W. Blair . 2021. Bases for the establishment of robusta coffee (*Coffea canephora*) as a new crop for Colombia. Agronomy 11: 2550.

[aps311584-bib-0013] Camus, M. F. , B. Alexander‐Lawrie , J. Sharbrough , and G. D. D. Hurst . 2022. Inheritance through the cytoplasm. Heredity 129: 31–43. 35525886 10.1038/s41437-022-00540-2 PMC9273588

[aps311584-bib-0014] Castresana, J. 2000. Selection of conserved blocks from multiple alignments for their use in phylogenetic analysis. Molecular Biology and Evolution 17: 540–552. 10742046 10.1093/oxfordjournals.molbev.a026334

[aps311584-bib-0015] Chalhoub, B. , F. Denoeud , S. Liu , I. A. P. Parkin , H. Tang , X. Wang , J. Chiquet , et al. 2014. Early allopolyploid evolution in the post‐Neolithic *Brassica napus* oilseed genome. Science 345: 950–953. 25146293 10.1126/science.1253435

[aps311584-bib-0016] Chen, J. , E. Li , X. Zhang , X. Dong , L. Lei , W. Song , H. Zhao , and J. Lai . 2017. Genome‐wide nucleosome occupancy and organization modulates the plasticity of gene transcriptional status in maize. Molecular Plant 10: 962–974. 28487258 10.1016/j.molp.2017.05.001

[aps311584-bib-0017] Chen, S. , F. Ren , L. Zhang , Y. Liu , X. Chen , Y. Li , L. Zhang , et al. 2018. Unstable allotetraploid tobacco genome due to frequent homeologous recombination, segmental deletion, and chromosome loss. Molecular Plant 11: 914–927. 29734001 10.1016/j.molp.2018.04.009

[aps311584-bib-0018] Chester, M. , J. P. Gallagher , V. V. Symonds , A. V. Cruz da Silva , E. V. Mavrodiev , A. R. Leitch , P. S. Soltis , and D. E. Soltis . 2012. Extensive chromosomal variation in a recently formed natural allopolyploid species, *Tragopogon miscellus* (Asteraceae). Proceedings of the National Academy of Sciences, USA 109: 1176–1181. 10.1073/pnas.1112041109 PMC3268322 22228301

[aps311584-bib-0019] Chu, Y. , D. Bertioli , C. M. Levinson , H. T. Stalker , C. C. Holbrook , and P. Ozias‐Akins . 2021. Homoeologous recombination is recurrent in the nascent synthetic allotetraploid *Arachis ipaënsis* × *Arachis correntina* ^4x^ and its derivatives. Genes, Genomes, Genetics 11: jkab066. 33693764 10.1093/g3journal/jkab066 PMC8759810

[aps311584-bib-0020] Clegg, M. T. , M. P. Cummings , and M. L. Durbin . 1997. The evolution of plant nuclear genes. Proceedings of the National Academy of Sciences, USA 94: 7791–7798. 10.1073/pnas.94.15.7791 PMC33705 9223265

[aps311584-bib-0021] Comai, L. 2005. The advantages and disadvantages of being polyploid. Nature Reviews Genetics 6: 836–846. 10.1038/nrg1711 16304599

[aps311584-bib-0022] Combes, M.‐C. , A. Dereeper , D. Severac , B. Bertrand , and P. Lashermes . 2013. Contribution of subgenomes to the transcriptome and their intertwined regulation in the allopolyploid *Coffea arabica* grown at contrasted temperatures. New Phytologist 200: 251–260. 23790161 10.1111/nph.12371

[aps311584-bib-0023] Conover, J. L. , and J. F. Wendel . 2022. Deleterious mutations accumulate faster in allopolyploid than diploid cotton (*Gossypium*) and unequally between subgenomes. Molecular Biology and Evolution 39: msac024. 35099532 10.1093/molbev/msac024 PMC8841602

[aps311584-bib-0024] Conover, J. L. , C. E. Grover , J. Sharbrough , D. B. Sloan , D. G. Peterson , and J. F. Wendel . 2023. Little evidence for homoeologous gene conversion and homoeologous exchange events in *Gossypium* allopolyploids. bioRxiv: 2023.11.08.566278 [preprint]. Available at 10.1101/2023.11.08.566278 [posted 12 November 2023; accessed 9 April 2024]. 39107998

[aps311584-bib-0025] Cros, J. , M. C. Combes , P. Trouslot , F. Anthony , S. Hamon , A. Charrier , and P. Lashermes . 1998. Phylogenetic analysis of chloroplast DNA variation in *Coffea* L. Molecular Phylogenetics and Evolution 9: 109–117. 9479700 10.1006/mpev.1997.0453

[aps311584-bib-0026] Deb, S. K. , P. P. Edger , J. C. Pires , and M. R. McKain . 2023. Patterns, mechanisms, and consequences of homoeologous exchange in allopolyploid angiosperms: A genomic and epigenomic perspective. New Phytologist 238: 2284–2304. 37010081 10.1111/nph.18927

[aps311584-bib-0027] Delabre, M. , N. El‐Mabrouk , K. T. Huber , M. Lafond , V. Moulton , E. Noutahi , and M. S. Castellanos . 2020. Evolution through segmental duplications and losses: A Super‐Reconciliation approach. Algorithms for Molecular Biology 15: 12. 32508979 10.1186/s13015-020-00171-4 PMC7249433

[aps311584-bib-0028] Ding, M. , and Z. J. Chen . 2018. Epigenetic perspectives on the evolution and domestication of polyploid plant and crops. Current Opinion in Plant Biology 42: 37–48. 29502038 10.1016/j.pbi.2018.02.003 PMC6058195

[aps311584-bib-0029] Douglas, G. M. , G. Gos , K. A. Steige , A. Salcedo , K. Holm , E. B. Josephs , R. Arunkumar , et al. 2015. Hybrid origins and the earliest stages of diploidization in the highly successful recent polyploid *Capsella bursa‐pastoris* . Proceedings of the National Academy of Sciences, USA 112: 2806–2811. 10.1073/pnas.1412277112 PMC4352811 25691747

[aps311584-bib-0030] Doyle, J. J. , and J. E. Coate . 2019. Polyploidy, the nucleotype, and novelty: The impact of genome doubling on the biology of the cell. International Journal of Plant Sciences 180: 1–52.

[aps311584-bib-0031] Doyle, J. J. , L. E. Flagel , A. H. Paterson , R. A. Rapp , D. E. Soltis , P. S. Soltis , and J. F. Wendel . 2008. Evolutionary genetics of genome merger and doubling in plants. Annual Review of Genetics 42: 443–461. 10.1146/annurev.genet.42.110807.091524 18983261

[aps311584-bib-0032] Drummond, D. A. , J. D. Bloom , C. Adami , C. O. Wilke , and F. H. Arnold . 2005. Why highly expressed proteins evolve slowly. Proceedings of the National Academy of Sciences, USA 102: 14338–14343. 10.1073/pnas.0504070102 PMC1242296 16176987

[aps311584-bib-0033] Durand, E. Y. , N. Patterson , D. Reich , and M. Slatkin . 2011. Testing for ancient admixture between closely related populations. Molecular Biology and Evolution 28: 2239–2252. 21325092 10.1093/molbev/msr048 PMC3144383

[aps311584-bib-0034] Edger, P. P. , R. Smith , M. R. McKain , and A. M. Cooley . 2017. Subgenome dominance in an interspecific hybrid, synthetic allopolyploid, and a 140‐year‐old naturally established neo‐allopolyploid monkeyflower. The Plant Cell 29(9): 2150–2167. 28814644 10.1105/tpc.17.00010 PMC5635986

[aps311584-bib-0035] Edwards, K. D. , N. Fernandez‐Pozo , K. Drake‐Stowe , M. Humphry , A. D. Evans , A. Bombarely , F. Allen , et al. 2017. A reference genome for *Nicotiana tabacum* enables map‐based cloning of homeologous loci implicated in nitrogen utilization efficiency. BMC Genomics 18: 448. 28625162 10.1186/s12864-017-3791-6 PMC5474855

[aps311584-bib-0036] Ellis, R. J. 1979. The most abundant protein in the world. Trends in Biochemical Sciences 4: 241–244.

[aps311584-bib-0037] Endrizzi, J. E. 1962. The diploid‐like cytological behavior of tetraploid cotton. Evolution 16: 325–329.

[aps311584-bib-0038] Feliner, G. N. , and J. A. Rosselló . 2012. Concerted evolution of multigene families and homoeologous recombination. *In* J. F. Wendel , J. Greilhuber , J. Dolezel , and I. J. Leitch [eds.], Plant Genome Diversity, Volume 1: Plant Genomes, their Residents, and their Evolutionary Dynamics, 171–193. Springer, Vienna, Austria.

[aps311584-bib-0039] Fernandes Gyorfy, M. , E. R. Miller , J. L. Conover , C. E. Grover , J. F. Wendel , D. B. Sloan , and J. Sharbrough . 2021. Nuclear‐cytoplasmic balance: Whole genome duplications induce elevated organellar genome copy number. The Plant Journal 108: 219–230. 34309123 10.1111/tpj.15436

[aps311584-bib-0040] Forsythe, E. S. , J. Sharbrough , J. C. Havird , J. M. Warren , and D. B. Sloan . 2019. CyMIRA: The cytonuclear molecular interactions reference for *Arabidopsis* . Genome Biology and Evolution 11: 2194–2202. 31282937 10.1093/gbe/evz144 PMC6685490

[aps311584-bib-0041] Forsythe, E. S. , A. D. L. Nelson , and M. A. Beilstein . 2020. Biased gene retention in the face of introgression obscures species relationships. Genome Biology and Evolution 12: 1646–1663. 33011798 10.1093/gbe/evaa149 PMC7533067

[aps311584-bib-0042] Forsythe, E. S. , C. E. Grover , E. R. Miller , J. L. Conover , M. A. Arick , M. C. F. Chavarro , S. C. M. Leal‐Bertioli , et al. 2022. Organellar transcripts dominate the cellular mRNA pool across plants of varying ploidy levels. Proceedings of the National Academy of Sciences, USA 119: e2204187119. 10.1073/pnas.2204187119 PMC9335225 35858449

[aps311584-bib-0043] Fox, D. T. , D. E. Soltis , P. S. Soltis , T.‐L. Ashman , and Y. Van de Peer . 2020. Polyploidy: A biological force from cells to ecosystems. Trends in Cell Biology 30: 688–694. 32646579 10.1016/j.tcb.2020.06.006 PMC7484144

[aps311584-bib-0044] Frankel, L. E. , and C. Ané . 2023. Summary tests of introgression are highly sensitive to rate variation across lineages. Systematic Biology 72: 1357–1369. 37698548 10.1093/sysbio/syad056

[aps311584-bib-0045] Fulneček, J. , R. Matyášek , and A. Kovařík . 2009. Faithful inheritance of cytosine methylation patterns in repeated sequences of the allotetraploid tobacco correlates with the expression of DNA methyltransferase gene families from both parental genomes. Molecular Genetics and Genomics 281: 407–420. 19132393 10.1007/s00438-008-0420-8

[aps311584-bib-0046] Gaeta, R. T. , J. C. Pires , F. Iniguez‐Luy , E. Leon , and T. C. Osborn . 2007. Genomic changes in resynthesized *Brassica napus* and their effect on gene expression and phenotype. The Plant Cell 19: 3403–3417. 18024568 10.1105/tpc.107.054346 PMC2174891

[aps311584-bib-0047] Gong, L. , A. Salmon , M.‐J. Yoo , K. K. Grupp , Z. Wang , A. H. Paterson , and J. F. Wendel . 2012. The cytonuclear dimension of allopolyploid evolution: An example from cotton using rubisco. Molecular Biology and Evolution 29: 3023–3036. 22490824 10.1093/molbev/mss110

[aps311584-bib-0048] Gong, L. , M. Olson , and J. F. Wendel . 2014. Cytonuclear evolution of Rubisco in four allopolyploid lineages. Molecular Biology and Evolution 31: 2624–2636. 25015644 10.1093/molbev/msu207 PMC4166922

[aps311584-bib-0049] Gonzalo, A. , P. Parra‐Nunez , A. L. Bachmann , E. Sanchez‐Moran , and K. Bomblies . 2023. Partial cytological diploidization of neoautotetraploid meiosis by induced cross‐over rate reduction. Proceedings of the National Academy of Sciences, USA 120: e2305002120. 10.1073/pnas.2305002120 PMC10434300 37549263

[aps311584-bib-0050] Gordon, S. P. , B. Contreras‐Moreira , J. J. Levy , A. Djamei , A. Czedik‐Eysenberg , V. S. Tartaglio , A. Session , et al. 2020. Gradual polyploid genome evolution revealed by pan‐genomic analysis of *Brachypodium hybridum* and its diploid progenitors. Nature Communications 11: 3670. 10.1038/s41467-020-17302-5 PMC7391716 32728126

[aps311584-bib-0051] Guo, H. , X. Wang , H. Gundlach , K. F. X. Mayer , D. G. Peterson , B. E. Scheffler , P. W. Chee , and A. H. Paterson . 2014. Extensive and biased intergenomic nonreciprocal DNA exchanges shaped a nascent polyploid genome, *Gossypium* (cotton). Genetics 197: 1153–1163. 24907262 10.1534/genetics.114.166124 PMC4125390

[aps311584-bib-0052] Hahn, M. W. , and M. S. Hibbins . 2019. A three‐sample test for introgression. Molecular Biology and Evolution 36: 2878–2882. 31373630 10.1093/molbev/msz178

[aps311584-bib-0053] Havird, J. C. , N. S. Whitehill , C. D. Snow , and D. B. Sloan . 2015. Conservative and compensatory evolution in oxidative phosphorylation complexes of angiosperms with highly divergent rates of mitochondrial genome evolution. Evolution; International Journal of Organic Evolution 69: 3069–3081. 26514987 10.1111/evo.12808 PMC4715514

[aps311584-bib-0054] Heslop‐Harrison, J. S. P. , T. Schwarzacher , and Q. Liu . 2023. Polyploidy: Its consequences and enabling role in plant diversification and evolution. Annals of Botany 131: 1–10. 36282971 10.1093/aob/mcac132 PMC9904344

[aps311584-bib-0055] Hibbins, M. S. , and M. W. Hahn . 2022. Corrigendum to: Phylogenomic approaches to detecting and characterizing introgression. Genetics 220: iyab220. 10.1093/genetics/iyab220 35100344 PMC8825298

[aps311584-bib-0056] Holm, S. 1979. A simple sequentially rejective multiple test procedure. Scandinavian Journal of Statistics, Theory and Applications 6: 65–70.

[aps311584-bib-0057] Hu, G. , R. Hovav , C. E. Grover , A. Faigenboim‐Doron , N. Kadmon , J. T. Page , J. A. Udall , and J. F. Wendel . 2016. Evolutionary conservation and divergence of gene coexpression networks in *Gossypium* (cotton) seeds. Genome Biology and Evolution 8: 3765–3783. 28062755 10.1093/gbe/evw280 PMC5585989

[aps311584-bib-0058] Jarvis, D. E. , Y. S. Ho , D. J. Lightfoot , S. M. Schmöckel , B. Li , T. J. A. Borm , H. Ohyanagi , et al. 2017. The genome of *Chenopodium quinoa* . Nature 542: 307–312. 28178233 10.1038/nature21370

[aps311584-bib-0059] Jiao, Y. , N. J. Wickett , S. Ayyampalayam , A. S. Chanderbali , L. Landherr , P. E. Ralph , L. P. Tomsho , et al. 2011. Ancestral polyploidy in seed plants and angiosperms. Nature 473: 97–100. 21478875 10.1038/nature09916

[aps311584-bib-0060] Katoh, K. , and D. M. Standley . 2013. MAFFT multiple sequence alignment software version 7: Improvements in performance and usability. Molecular Biology and Evolution 30: 772–780. 23329690 10.1093/molbev/mst010 PMC3603318

[aps311584-bib-0061] Kihara, H. , and T. Ono . 1926. Chromosomenzahlen und systematische Gruppierung der Rumex‐Arten. Zeitschrift für Zellforschung und Mikroskopische Anatomie 4: 475–481.

[aps311584-bib-0062] Kovarik, A. , J. C. Pires , A. R. Leitch , K. Y. Lim , A. M. Sherwood , R. Matyasek , J. Rocca , et al. 2005. Rapid concerted evolution of nuclear ribosomal DNA in two *Tragopogon* allopolyploids of recent and recurrent origin. Genetics 169: 931–944. 15654116 10.1534/genetics.104.032839 PMC1449095

[aps311584-bib-0063] Kremnev, D. , and A. Strand . 2014. Plastid encoded RNA polymerase activity and expression of photosynthesis genes required for embryo and seed development in *Arabidopsis* . Frontiers in Plant Science 5: 385. 25161659 10.3389/fpls.2014.00385 PMC4130184

[aps311584-bib-0064] Kühn, K. , T. Obata , K. Feher , R. Bock , A. R. Fernie , and E. H. Meyer . 2015. Complete mitochondrial complex I deficiency induces an up‐regulation of respiratory fluxes that is abolished by traces of functional complex I. Plant Physiology 168: 1537–1549. 26134164 10.1104/pp.15.00589 PMC4528760

[aps311584-bib-0065] Landis, J. B. , A. Kurti , A. J. Lawhorn , A. Litt , and E. W. McCarthy . 2020. Differential gene expression with an emphasis on floral organ size differences in natural and synthetic polyploids of *Nicotiana tabacum* (Solanaceae). Genes 11(9): 1097. 32961813 10.3390/genes11091097 PMC7563459

[aps311584-bib-0066] Lashermes, P. , M. C. Combes , J. Robert , P. Trouslot , A. D'Hont , F. Anthony , and A. Charrier . 1999. Molecular characterisation and origin of the *Coffea arabica* L. genome. Molecular and General Genetics 261: 259–266. 10102360 10.1007/s004380050965

[aps311584-bib-0067] Lashermes, P. , Y. Hueber , M.‐C. Combes , D. Severac , and A. Dereeper . 2016. Inter‐genomic DNA exchanges and homeologous gene silencing shaped the nascent allopolyploid coffee genome (*Coffea arabica* L.). Genes, Genomes, Genetics 6: 2937–2948. 27440920 10.1534/g3.116.030858 PMC5015950

[aps311584-bib-0068] Leal‐Bertioli, S. C. M. , I. J. Godoy , J. F. Santos , J. J. Doyle , P. M. Guimarães , B. L. Abernathy , S. A. Jackson , et al. 2018. Segmental allopolyploidy in action: Increasing diversity through polyploid hybridization and homoeologous recombination. American Journal of Botany 105: 1053–1066. 29985538 10.1002/ajb2.1112

[aps311584-bib-0069] Leitch, A. R. , and I. J. Leitch . 2008. Genomic plasticity and the diversity of polyploid plants. Science 320: 481–483. 18436776 10.1126/science.1153585

[aps311584-bib-0070] Li, C. , X. Wang , Y. Xiao , X. Sun , J. Wang , X. Yang , Y. Sun , et al. 2020. Co‐evolution in hybrid genomes: Nuclear‐encoded Rubisco small subunits and their plastid‐targeting translocons accompanying sequential allopolyploidy events in *Triticum* . Molecular Biology and Evolution 37(12): 3409–3422. 32602899 10.1093/molbev/msaa158 PMC7743682

[aps311584-bib-0071] Madlung, A. , R. W. Masuelli , B. Watson , S. H. Reynolds , J. Davison , and L. Comai . 2002. Remodeling of DNA methylation and phenotypic and transcriptional changes in synthetic *Arabidopsis* allotetraploids. Plant Physiology 129: 733–746. 12068115 10.1104/pp.003095 PMC161697

[aps311584-bib-0072] Martin, S. H. , J. W. Davey , and C. D. Jiggins . 2014. Evaluating the use of ABBA–BABA statistics to locate introgressed loci. Molecular Biology and Evolution 32: 244–257. 25246699 10.1093/molbev/msu269 PMC4271521

[aps311584-bib-0073] Mason, A. S. , and J. C. Pires . 2015. Unreduced gametes: Meiotic mishap or evolutionary mechanism? Trends in Genetics 31: 5–10. 25445549 10.1016/j.tig.2014.09.011

[aps311584-bib-0074] Mason, A. S. , and J. F. Wendel . 2020. Homoeologous exchanges, segmental allopolyploidy, and polyploid genome evolution. Frontiers in Genetics 11: 1014. 33005183 10.3389/fgene.2020.01014 PMC7485112

[aps311584-bib-0075] Meagher, R. B. , S. Berry‐Lowe , and K. Rice . 1989. Molecular evolution of the small subunit of ribulose bisphosphate carboxylase: Nucleotide substitution and gene conversion. Genetics 123: 845–863. 2515110 10.1093/genetics/123.4.845 PMC1203894

[aps311584-bib-0076] Mekbib, Y. , K. Tesfaye , X. Dong , J. K. Saina , G.‐W. Hu , and Q.‐F. Wang . 2022. Whole‐genome resequencing of *Coffea arabica* L. (Rubiaceae) genotypes identify SNP and unravels distinct groups showing a strong geographical pattern. BMC Plant Biology 22: 69. 35164709 10.1186/s12870-022-03449-4 PMC8842891

[aps311584-bib-0077] Mirarab, S. , R. Reaz , M. S. Bayzid , T. Zimmermann , M. S. Swenson , and T. Warnow . 2014. ASTRAL: Genome‐scale coalescent‐based species tree estimation. Bioinformatics 30: i541–i548. 25161245 10.1093/bioinformatics/btu462 PMC4147915

[aps311584-bib-0078] Morel, B. , A. M. Kozlov , A. Stamatakis , and G. J. Szöllősi . 2020. GeneRax: A tool for species‐tree‐aware maximum likelihood‐based gene family tree inference under gene duplication, transfer, and loss. Molecular Biology and Evolution 37: 2763–2774. 32502238 10.1093/molbev/msaa141 PMC8312565

[aps311584-bib-0079] Nei, M. , and A. P. Rooney . 2005. Concerted and birth‐and‐death evolution of multigene families. Annual Review of Genetics 39: 121–152. 10.1146/annurev.genet.39.073003.112240 PMC1464479 16285855

[aps311584-bib-0080] Oberprieler, C. , M. Talianova , and J. Griesenbeck . 2019. Effects of polyploidy on the coordination of gene expression between organellar and nuclear genomes in *Leucanthemum* Mill. (Compositae, Anthemideae). Ecology and Evolution 9: 9100–9110. 31463007 10.1002/ece3.5455 PMC6706232

[aps311584-bib-0081] One Thousand Plant Transcriptomes Initiative . 2019. One thousand plant transcriptomes and the phylogenomics of green plants. Nature 574: 679–685. 31645766 10.1038/s41586-019-1693-2 PMC6872490

[aps311584-bib-0082] Orantes‐Bonilla, M. , M. Makhoul , H. Lee , H. S. Chawla , P. Vollrath , A. Langstroff , F. J. Sedlazeck , et al. 2022. Frequent spontaneous structural rearrangements promote rapid genome diversification in a *Brassica napus* F1 generation. Frontiers in Plant Science 13: 1057953. 36466276 10.3389/fpls.2022.1057953 PMC9716091

[aps311584-bib-0083] Oruganti, V. , H. Toegelová , A. Pečinka , A. Madlung , and K. Schneeberger . 2023. Rapid large‐scale genomic introgression in *Arabidopsis suecica* via an autoallohexaploid bridge. Genetics 223: iyac132. 36124968 10.1093/genetics/iyac132 PMC9910397

[aps311584-bib-0084] Otto, S. P. 2007. The evolutionary consequences of polyploidy. Cell 131: 452–462. 17981114 10.1016/j.cell.2007.10.022

[aps311584-bib-0085] Otto, S. P. , and J. Whitton . 2000. Polyploid incidence and evolution. Annual Review of Genetics 34: 401–437. 10.1146/annurev.genet.34.1.401 11092833

[aps311584-bib-0086] Parey, E. , A. Louis , C. Cabau , Y. Guiguen , H. Roest Crollius , and C. Berthelot . 2020. Synteny‐guided resolution of gene trees clarifies the functional impact of whole‐genome duplications. Molecular Biology and Evolution 37: 3324–3337. 32556216 10.1093/molbev/msaa149

[aps311584-bib-0087] Pont, C. , T. Leroy , M. Seidel , A. Tondelli , W. Duchemin , D. Armisen , D. Lang , et al. 2019. Tracing the ancestry of modern bread wheats. Nature Genetics 51: 905–911. 31043760 10.1038/s41588-019-0393-z

[aps311584-bib-0088] R Core Team . 2023. R: a language and environment for statistical computing. R Foundation for Statistical Computing, Vienna, Austria. Website: http://www.R-project.org [accessed 9 April 2024].

[aps311584-bib-0089] Ramírez‐González, R. H. , P. Borrill , D. Lang , S. A. Harrington , J. Brinton , L. Venturini , M. Davey , et al. 2018. The transcriptional landscape of polyploid wheat. Science 361: eaar6089. 30115782 10.1126/science.aar6089

[aps311584-bib-0090] Ramsey, J. , and D. W. Schemske . 2003. Neopolyploidy in flowering plants. Annual Review of Ecology, Evolution and Systematics 33: 589–639.

[aps311584-bib-0091] Rand, D. M. , R. A. Haney , and A. J. Fry . 2004. Cytonuclear coevolution: The genomics of cooperation. Trends in Ecology & Evolution 19: 645–653. 16701327 10.1016/j.tree.2004.10.003

[aps311584-bib-0092] Rao, X. , J. Ren , W. Wang , R. Chen , Q. Xie , Y. Xu , D. Li , et al. 2023. Comparative DNA‐methylome and transcriptome analysis reveals heterosis‐ and polyploidy‐associated epigenetic changes in rice. The Crop Journal 11: 427–437.

[aps311584-bib-0093] Raven, J. A. 2013. Rubisco: Still the most abundant protein of Earth? New Phytologist 198: 1–3. 23432200 10.1111/nph.12197

[aps311584-bib-0094] Rockenbach, K. , J. C. Havird , J. G. Monroe , D. A. Triant , D. R. Taylor , and D. B. Sloan . 2016. Positive selection in rapidly evolving plastid‐nuclear enzyme complexes. Genetics 204: 1507–1522. 27707788 10.1534/genetics.116.188268 PMC5161282

[aps311584-bib-0095] Román‐Palacios, C. , C. A. Medina , S. H. Zhan , and M. S. Barker . 2021. Animal chromosome counts reveal a similar range of chromosome numbers but with less polyploidy in animals compared to flowering plants. Journal of Evolutionary Biology 34: 1333–1339. 34101952 10.1111/jeb.13884

[aps311584-bib-0096] Ruprecht, C. , R. Lohaus , K. Vanneste , M. Mutwil , Z. Nikoloski , Y. Van de Peer , and S. Persson . 2017. Revisiting ancestral polyploidy in plants. Science Advances 3: e1603195. 28695205 10.1126/sciadv.1603195 PMC5498109

[aps311584-bib-0097] Salmon, A. , M. L. Ainouche , and J. F. Wendel . 2005. Genetic and epigenetic consequences of recent hybridization and polyploidy in *Spartina* (Poaceae). Molecular Ecology 14: 1163–1175. 15773943 10.1111/j.1365-294X.2005.02488.x

[aps311584-bib-0098] Salmon, A. , L. Flagel , B. Ying , J. A. Udall , and J. F. Wendel . 2010. Homoeologous nonreciprocal recombination in polyploid cotton. New Phytologist 186: 123–134. 19925554 10.1111/j.1469-8137.2009.03093.x

[aps311584-bib-0099] Salojarvi, J. , A. Rambani , Z. Yu , R. Guyot , S. Strickler , M. Lepelley , C. Wang , et al. 2023. The genome and population genomics of allopolyploid *Coffea arabica* reveal the diversification history of modern coffee cultivars. bioRxiv 2023.09.06.556570 [preprint]. Available at 10.1101/2023.09.06.556570 [posted 11 November 2023; accessed 9 April 2024]. PMC11018527 38622339

[aps311584-bib-0100] Scalabrin, S. , L. Toniutti , G. Di Gaspero , D. Scaglione , G. Magris , M. Vidotto , S. Pinosio , et al. 2020. A single polyploidization event at the origin of the tetraploid genome of *Coffea arabica* is responsible for the extremely low genetic variation in wild and cultivated germplasm. Scientific Reports 10: 4642. 32170172 10.1038/s41598-020-61216-7 PMC7069947

[aps311584-bib-0101] Schiessl, S.‐V. , E. Katche , E. Ihien , H. S. Chawla , and A. S. Mason . 2019. The role of genomic structural variation in the genetic improvement of polyploid crops. The Crop Journal 7: 127–140.

[aps311584-bib-0102] Schnable, J. C. , B. S. Pedersen , S. Subramaniam , and M. Freeling . 2011. Dose–sensitivity, conserved non‐coding sequences, and duplicate gene retention through multiple tetraploidies in the grasses. Frontiers in Plant Science 2: 2. 10.3389/fpls.2011.00002 22645525 PMC3355796

[aps311584-bib-0103] Sharbrough, J. , J. L. Conover , J. A. Tate , J. F. Wendel , and D. B. Sloan . 2017. Cytonuclear responses to genome doubling. American Journal of Botany 104: 1277–1280. 29885242 10.3732/ajb.1700293

[aps311584-bib-0104] Sharbrough, J. , J. L. Conover , M. Fernandes Gyorfy , C. E. Grover , E. R. Miller , J. F. Wendel , and D. B. Sloan . 2022. Global patterns of subgenome evolution in organelle‐targeted genes of six allotetraploid angiosperms. Molecular Biology and Evolution 39(4): msac074. 35383845 10.1093/molbev/msac074 PMC9040051

[aps311584-bib-0105] Shcherban, A. B. , E. D. Badaeva , A. V. Amosova , I. G. Adonina , and E. A. Salina . 2008. Genetic and epigenetic changes of rDNA in a synthetic allotetraploid, *Aegilops sharonensis* × *Ae. umbellulata* . Genome 51: 261–271. 18356962 10.1139/G08-006

[aps311584-bib-0106] Sloan, D. B. , J. C. Havird , and J. Sharbrough . 2017. The on‐again, off‐again relationship between mitochondrial genomes and species boundaries. Molecular Ecology 26: 2212–2236. 27997046 10.1111/mec.13959 PMC6534505

[aps311584-bib-0107] Sloan, D. B. , J. M. Warren , A. M. Williams , Z. Wu , S. E. Abdel‐Ghany , A. J. Chicco , and J. C. Havird . 2018. Cytonuclear integration and co‐evolution. Nature Reviews Genetics 19: 635–648. 10.1038/s41576-018-0035-9 PMC6469396 30018367

[aps311584-bib-0108] Soltis, D. E. , C. J. Visger , and P. S. Soltis . 2014. The polyploidy revolution then… and now: Stebbins revisited. American Journal of Botany 101: 1057–1078. 25049267 10.3732/ajb.1400178

[aps311584-bib-0109] Song, B. , S. Marco‐Sola , M. Moreto , L. Johnson , E. S. Buckler , and M. C. Stitzer . 2022. AnchorWave: Sensitive alignment of genomes with high sequence diversity, extensive structural polymorphism, and whole‐genome duplication. Proceedings of the National Academy of Sciences, USA 119: e2113075119. 10.1073/pnas.2113075119 PMC8740769 34934012

[aps311584-bib-0110] Song, M. J. , B. I. Potter , J. J. Doyle , and J. E. Coate . 2020. Gene balance predicts transcriptional responses immediately following ploidy change in *Arabidopsis thaliana* . The Plant Cell 32: 1434–1448. 32184347 10.1105/tpc.19.00832 PMC7203931

[aps311584-bib-0111] Song, Q. , T. Zhang , D. M. Stelly , and Z. J. Chen . 2017. Epigenomic and functional analyses reveal roles of epialleles in the loss of photoperiod sensitivity during domestication of allotetraploid cottons. Genome Biology 18: 99. 28558752 10.1186/s13059-017-1229-8 PMC5450403

[aps311584-bib-0112] Stamatakis, A. 2014. RAxML version 8: A tool for phylogenetic analysis and post‐analysis of large phylogenies. Bioinformatics 30: 1312–1313. 24451623 10.1093/bioinformatics/btu033 PMC3998144

[aps311584-bib-0113] Steenwyk, J. L. , T. J. Buida , Y. Li , X.‐X. Shen , and A. Rokas . 2020. ClipKIT: A multiple sequence alignment trimming software for accurate phylogenomic inference. PLoS Biology 18: e3001007. 33264284 10.1371/journal.pbio.3001007 PMC7735675

[aps311584-bib-0114] Stein, A. , O. Coriton , M. Rousseau‐Gueutin , B. Samans , S. V. Schiessl , C. Obermeier , I. A. P. Parkin , et al. 2017. Mapping of homoeologous chromosome exchanges influencing quantitative trait variation in *Brassica napus* . Plant Biotechnology Journal 15: 1478–1489. 28370938 10.1111/pbi.12732 PMC5633767

[aps311584-bib-0115] Wendel, J. F. 2015. The wondrous cycles of polyploidy in plants. American Journal of Botany 102: 1753–1756. 26451037 10.3732/ajb.1500320

[aps311584-bib-0116] Wendel, J. F. , S. A. Jackson , B. C. Meyers , and R. A. Wing . 2016. Evolution of plant genome architecture. Genome Biology 17: 37. 26926526 10.1186/s13059-016-0908-1 PMC4772531

[aps311584-bib-0117] Wickham, H. 2011. ggplot2. Wiley Interdisciplinary Reviews: Computational Statistics 3: 180–185.

[aps311584-bib-0118] Williams, A. M. , G. Friso , K. J. van Wijk , and D. B. Sloan . 2019. Extreme variation in rates of evolution in the plastid Clp protease complex. The Plant Journal 98: 243–259. 30570818 10.1111/tpj.14208

[aps311584-bib-0119] Willson, J. , M. S. Roddur , B. Liu , P. Zaharias , and T. Warnow . 2022. DISCO: Species tree inference using multicopy gene family tree decomposition. Systematic Biology 71: 610–629. 34450658 10.1093/sysbio/syab070 PMC9016570

[aps311584-bib-0120] Wolfe, K. H. , W. H. Li , and P. M. Sharp . 1987. Rates of nucleotide substitution vary greatly among plant mitochondrial, chloroplast, and nuclear DNAs. Proceedings of the National Academy of Sciences, USA 84: 9054–9058. 10.1073/pnas.84.24.9054 PMC299690 3480529

[aps311584-bib-0121] Xiong, Z. , R. T. Gaeta , P. P. Edger , Y. Cao , K. Zhao , S. Zhang , and J. C. Pires . 2020. Chromosome inheritance and meiotic stability in allopolyploid *Brassica napus* . Genes, Genomes, Genetics 11(2): jkaa011. 10.1093/g3journal/jkaa011 PMC8022990 33704431

[aps311584-bib-0122] Yang, J. , D. Liu , X. Wang , C. Ji , F. Cheng , B. Liu , Z. Hu , et al. 2016. The genome sequence of allopolyploid *Brassica juncea* and analysis of differential homoeolog gene expression influencing selection. Nature Genetics 48: 1225–1232. 27595476 10.1038/ng.3657

[aps311584-bib-0123] Yang, J.‐R. , B.‐Y. Liao , S.‐M. Zhuang , and J. Zhang . 2012. Protein misinteraction avoidance causes highly expressed proteins to evolve slowly. Proceedings of the National Academy of Sciences, USA 109: E831–E840. 10.1073/pnas.1117408109 PMC3325723 22416125

[aps311584-bib-0124] Yoo, M.‐J. , E. Szadkowski , and J. F. Wendel . 2013. Homoeolog expression bias and expression level dominance in allopolyploid cotton. Heredity 110: 171–180. 23169565 10.1038/hdy.2012.94 PMC3554454

[aps311584-bib-0125] Yuan, Y. , Z. Milec , P. E. Bayer , J. Vrána , J. Doležel , D. Edwards , W. Erskine , and P. Kaur . 2018. Large‐scale structural variation detection in subterranean clover subtypes using optical mapping. Frontiers in Plant Science 9: 971. 30065731 10.3389/fpls.2018.00971 PMC6056659

[aps311584-bib-0126] Zhang, Z. , X. Gou , H. Xun , Y. Bian , X. Ma , J. Li , N. Li , et al. 2020. Homoeologous exchanges occur through intragenic recombination generating novel transcripts and proteins in wheat and other polyploids. Proceedings of the National Academy of Sciences, USA 117: 14561–14571. 10.1073/pnas.2003505117 PMC7321986 32518116

[aps311584-bib-0127] Zimmer, E. A. , S. L. Martin , S. M. Beverley , Y. W. Kan , and A. C. Wilson . 1980. Rapid duplication and loss of genes coding for the alpha chains of hemoglobin. Proceedings of the National Academy of Sciences, USA 77: 2158–2162. 10.1073/pnas.77.4.2158 PMC348671 6929543

