# Supplementary material for: Genome‐wide patterns of homoeologous gene flow in allotetraploid coffee
Source: Appl Plant Sci. 2024 Jun 14;12(4):e11584. doi: 10.1002/aps3.11584 (PMC11342229; doi:10.1002/aps3.11584)
Supplement: Supplementary file 1 — Appendix S1. Graphical depiction of the classic implementation of the ABBA‐BABA test. The relative abundance of ABBA (middle tree) vs. BABA (right tree) site patterns in genome‐wide alignments can be used to infer introgression between species in a four‐taxon arrangement. [file APS3-12-e11584-s005.pdf]

DOI 10.1002/aps3.11584

APPENDIX S1. Graphical depiction of the classic implementation of the ABBA-BABA test. The relative abundance of ABBA (middle tree) vs. BABA (right tree) site patterns in genome-wide alignments can be used to infer introgression between species in a four-taxon arrangement.

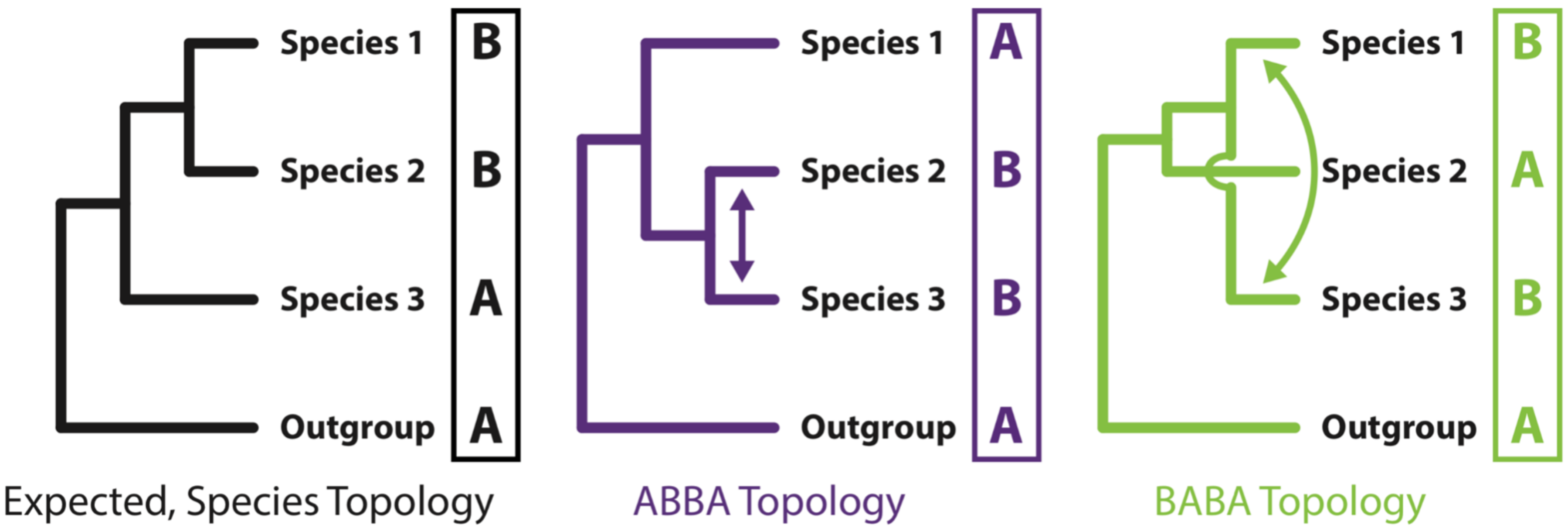

**Cannot distinguish from *I.L.S.* :**	=	**BABA**
**Introgression between S3 and S2:**	>	**BABA**
**Introgression between S3 and S1:**	<	**BABA**
