# Supplementary material for: Genome‐wide patterns of homoeologous gene flow in allotetraploid coffee
Source: Appl Plant Sci. 2024 Jun 14;12(4):e11584. doi: 10.1002/aps3.11584 (PMC11342229; doi:10.1002/aps3.11584)

DOI 10.1002/aps3.11584

APPENDIX S3. Patterns of HGF in genome-wide and mitochondria- and plastid-targeted genes using Gblocks-trimmed alignments in allotetraploid *Coffea arabica*. Reciprocal *D*-statistic estimates from Gblocks-trimmed alignments of the E subgenome overwriting the C subgenome (purple, D_{MAT}) and of the C subgenome overwriting the E subgenome (green, D_{PAT}) in all genes (top panel) and in genes targeted to the mitochondria (left) and to the plastid (right). Genes are grouped according to the intimacy of interaction: non-interacting – top panels, interacting – middle panels, enzyme complexes – bottom panels. Points represent overall *D*-statistics, density plots depict distributions from 10,000 gene-level bootstrap replicates, and error bars represent 95% CIs. Distributions that are significantly greater than 0 are indicative of HGF in that direction.

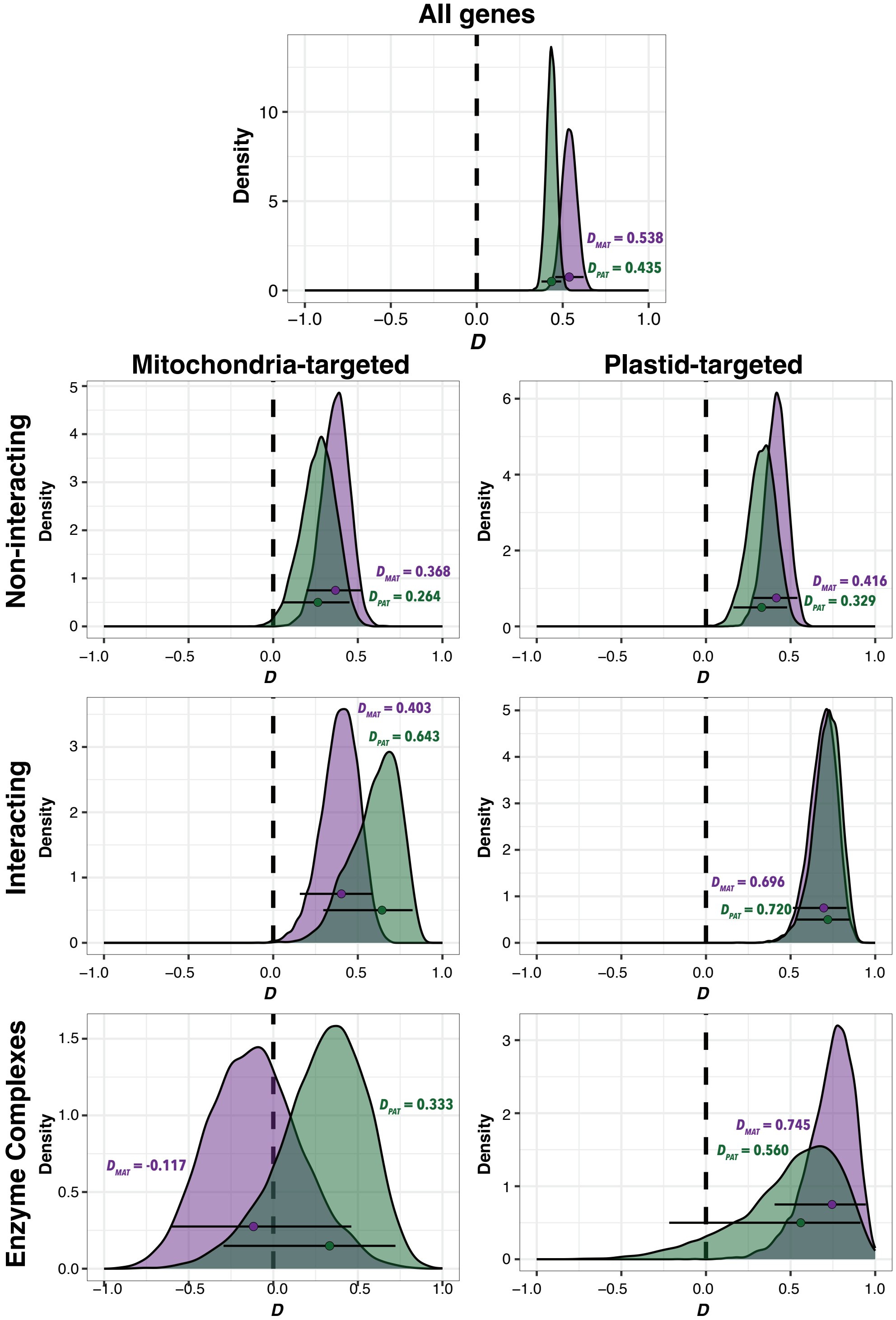

Supplement: Supplementary file 3 — Appendix S3. Patterns of HGF in genome‐wide and mitochondria‐ and plastid‐targeted genes using Gblocks‐trimmed alignments in allotetraploid Coffea arabica. Reciprocal D‐statistic estimates from Gblocks‐trimmed alignments of the E subgenome overwriting the C subgenome (purple, D MAT ) and of the C subgenome overwriting the E subgenome (green, D PAT ) in all genes (top panel) and in genes targeted to the mitochondria (left) and to the plastid (right). Genes are grouped according to the intimacy of interaction: non‐interacting – top panels, interacting – middle panels, enzyme complexes – bottom panels. Points represent overall D‐statistics, density plots depict distributions from 10,000 gene‐level bootstrap replicates, and error bars represent 95% CIs. Distributions that are significantly greater than 0 are indicative of HGF in that direction. [file APS3-12-e11584-s009.pdf]
