# Supplementary material for: Genome‐wide patterns of homoeologous gene flow in allotetraploid coffee
Source: Appl Plant Sci. 2024 Jun 14;12(4):e11584. doi: 10.1002/aps3.11584 (PMC11342229; doi:10.1002/aps3.11584)

DOI 10.1002/aps3.11584

APPENDIX S5. Graphical depiction of the possible gene tree topologies for rooted, five-taxon trees. There are 15 possible tree topologies for a rooted tree with five taxa. The *Coffea* species tree is depicted in (A) (highlighted in gray). The gene tree that would be expected if HGF were reciprocal across subgenomes is depicted in (F) (highlighted in orange). The gene tree that would be expected if HGF were maternally biased (i.e., E-overwriting-C) is depicted in (L) (highlighted in purple). The gene tree that would be expected if HGF were paternally biased (i.e., C-overwriting-E) is depicted in (M) (highlighted in green).

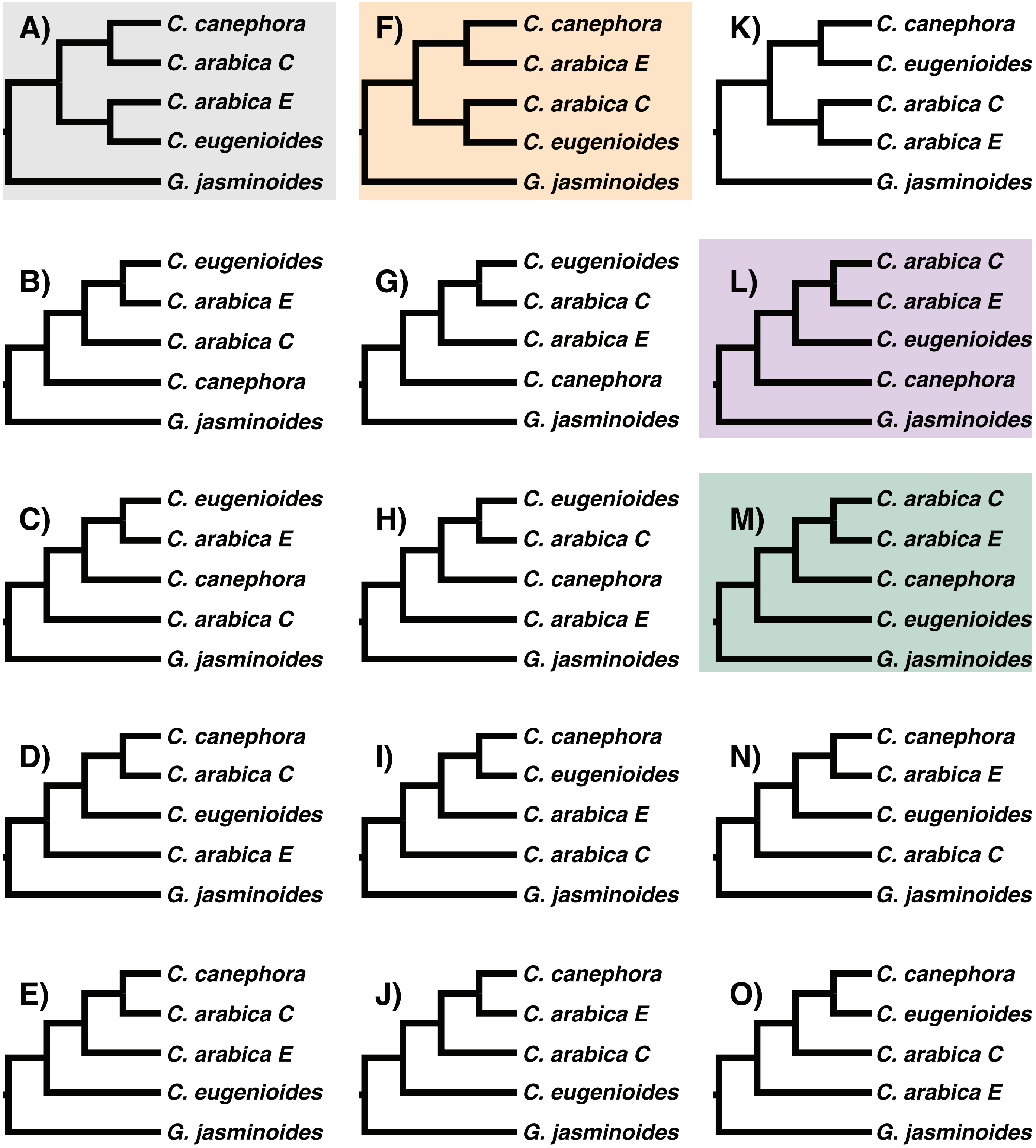

Supplement: Supplementary file 5 — Appendix S5. Graphical depiction of the possible gene tree topologies for rooted, five‐taxon trees. There are 15 possible tree topologies for a rooted tree with five taxa. The Coffea species tree is depicted in (A) (highlighted in gray). The gene tree that would be expected if HGF were reciprocal across subgenomes is depicted in (F) (highlighted in orange). The gene tree that would be expected if HGF were maternally biased (i.e., E‐overwriting‐C) is depicted in (L) (highlighted in purple). The gene tree that would be expected if HGF were paternally biased (i.e., C‐overwriting‐E) is depicted in (M) (highlighted in green). [file APS3-12-e11584-s003.pdf]
