# Supplementary material for: Genome‐wide patterns of homoeologous gene flow in allotetraploid coffee
Source: Appl Plant Sci. 2024 Jun 14;12(4):e11584. doi: 10.1002/aps3.11584 (PMC11342229; doi:10.1002/aps3.11584)

DOI 10.1002/aps3.11584

APPENDIX S6. Graphical depiction of species tree, HGF trees, and ILS tree topologies, showing the comparison between putative HGF gene trees with alternative gene tree topologies that are due to random sorting of alleles, recurrent mutations, and autapomorphies. See Appendix S5 for the possible gene tree topologies.

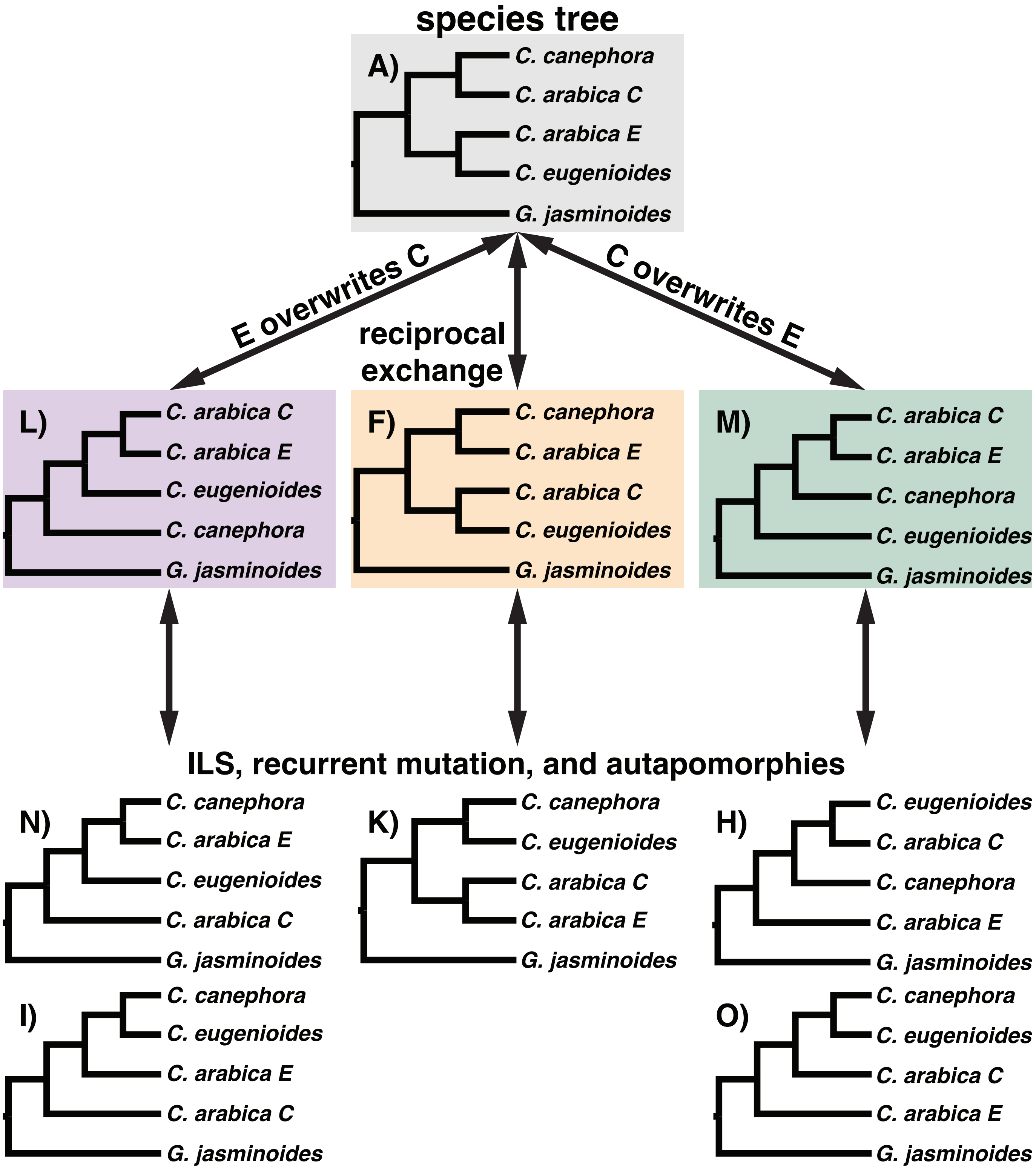

Supplement: Supplementary file 6 — Appendix S6. Graphical depiction of species tree, HGF trees, and ILS tree topologies, showing the comparison between putative HGF gene trees with alternative gene tree topologies that are due to random sorting of alleles, recurrent mutations, and autapomorphies. See Appendix S5 for the possible gene tree topologies. [file APS3-12-e11584-s001.pdf]
